# Chronic Administration of Carvone-Rich *Mentha spicata* L. Essential Oil Attenuates Cognitive Dysfunction and Oxidative Stress in Zebrafish

**DOI:** 10.3390/plants15182763

**Published:** 2026-09-09

**Authors:** Ion Brinza, Razvan Stefan Boiangiu, Eyup Bagci, Elena Todirascu-Ciornea, Lucian Hritcu, Gabriela Dumitru

**Affiliations:** 1 Department of Environmental Sciences, Biology and Ecology Research Center, Faculty of Sciences, “Lucian Blaga” University of Sibiu, Doctor. Ion Ratiu Street No. 5–7, 550012 Sibiu, Romania; ion.brinza@ulbsibiu.ro; 2 Department of Biology, Faculty of Biology, Alexandru Ioan Cuza University of Iasi, 700506 Iasi, Romania; razvan.boiangiu@uaic.ro (R.S.B.); ciornea@uaic.ro (E.T.-C.); gabriela.dumitru@uaic.ro (G.D.); 3 Department of Biology, Faculty of Sciences, Fırat University, 23119 Elazig, Turkey; ebagci@firat.edu.tr

**Keywords:** *Mentha spicata* essential oil, cognitive dysfunction, oxidative stress, scopolamine, zebrafish

## Abstract

Cognitive impairment and anxiety-like behavior associated with cholinergic dysfunction and oxidative stress remain important experimental targets for neuropharmacological screening. *Mentha spicata* essential oil (MEO, 150 or 300 µL/L) was administered daily by immersion from experimental day 1 through day 22; the first behavioral assessment occurred after 7 days of pre-exposure. Scopolamine hydrobromide trihydrate (SCO, 100 µM) was administered for 30 min before each behavioral assessment and again before euthanasia, whereas galantamine (GAL, 1 mg/L) was administered 30 min before each SCO challenge. Behavior was evaluated using the novel tank diving test (NTT), novel approach test (NAT), Y-maze, and novel object recognition test (NOR). Brain acetylcholinesterase (AChE) activity, antioxidant defenses, and oxidative damage were assessed. GC-MS identified a carvone-rich profile dominated by carvone (67.8%), limonene (10.6%), and 1,8-cineole (4.2%). MEO attenuated several SCO-associated behavioral and biochemical changes. The 150 µL/L concentration showed the most consistent memory-related improvement, whereas 300 µL/L also reduced selected oxidative markers but was associated with reduced exploratory activity in some tasks. Treatment-adjusted behavior–biomarker analyses were considered exploratory; only the inverse association between malondialdehyde (MDA) and the NTT top/bottom ratio remained significant after false-discovery-rate correction. In silico descriptors were treated as hypothesis-generating and do not demonstrate zebrafish brain exposure. These findings support further investigation of carvone-rich MEO within a rescue/protection framework, with confirmation in independently replicated exposure tanks and inclusion of MEO-only controls.

## 1. Introduction

Neurodegenerative diseases, especially Alzheimer’s disease (AD), are rising globally and remain without curative treatment. Their hallmark features, cognitive decline and behavioral disturbances, are driven by oxidative stress, neuroinflammation, and cholinergic dysfunction. Disruption of redox balance promotes neuronal damage, while altered acetylcholine (ACh) signaling worsens memory impairment [1,2]. These convergent mechanisms have generated growing interest in natural products with antioxidant, anti-inflammatory, and neuromodulatory properties as potential adjuvant strategies [3,4,5].

The zebrafish (*Danio rerio*) is a validated vertebrate model for studying cognition and anxiety, enabling high-throughput behavioral phenotyping and brain biochemical analyses [6,7,8]. Pharmacological disruption of cholinergic tone with scopolamine hydrobromide trihydrate (SCO), a muscarinic antagonist, consistently induces memory deficits, anxiety-like behavior, and redox imbalance. Galantamine (GAL), a clinically used acetylcholinesterase (AChE) inhibitor in AD, is commonly employed as a positive control because it restores performance and mitigates oxidative disturbances [9,10].

In this context, essential oils from *Mentha* species (MEO), rich in monoterpenes [11,12], are typically dominated by carvone as the principal constituent, accompanied by minor dihydrocarvone derivatives. This compositional signature is consistent with reports on *M. spicata* oils from Turkey/Anatolia, where carvone is frequently observed in the ~59–77% range, alongside variable contributions of limonene (up to ~23%), 1,8-cineole (~1–7%), and trans-dihydrocarvone (~1–4%) [13]. Beyond the antioxidant and anti-inflammatory profile described for carvone-rich *M. spicata* oils, the dominant monoterpenes also exhibit activities relevant to the central nervous system (CNS): carvone has been associated with cholinesterase-inhibitory potential and interactions at GABAA receptors, whereas limonene may modulate dopaminergic/GABAergic function; additionally, 1,8-cineole has shown antioxidant and neuroprotective effects linked to redox-related mechanisms [14]. However, despite the increasingly common use of chronic immersion exposure paradigms with essential oils in the SCO-treated zebrafish model, the impact of prolonged exposure to a carvone-dominant *Mentha* oil on SCO-induced behavioral deficits and brain oxidative imbalance in *D. rerio* remains insufficiently characterized.

Direct experimental evidence for CNS exposure should be distinguished from computational prediction. Independent in vivo/ex vivo work has detected S-(+)-carvone in brain tissue, including the hippocampus, providing empirical evidence that this major spearmint monoterpene can reach the CNS [15]. More generally, experimental blood–brain barrier studies show that passive brain uptake is governed by a combination of lipophilicity, molecular size, polarity, ionization, and membrane/transport interactions rather than by hydrogen-bonding capacity or topological polar surface area (TPSA) alone [16]. No brain concentration or pharmacokinetic measurements were performed for MEO constituents in the present zebrafish experiment; consequently, the computational analyses described below are used only as exploratory, hypothesis-generating context and not as evidence of compound uptake.

In this study, we evaluated whether chronic MEO exposure (150 or 300 µL/L) attenuates SCO-associated anxiety-like and memory-related alterations and brain oxidative imbalance, using GAL as a pharmacological positive control. MEO administration began on experimental day 1 and continued daily through day 22, while SCO was administered acutely before each behavioral assessment and again before tissue collection. The experimental assessment combined the novel tank diving test (NTT), novel approach test (NAT), Y-maze, and novel object recognition (NOR) test with measurements of acetylcholinesterase (AChE), superoxide dismutase (SOD), catalase (CAT), glutathione peroxidase (GPX), reduced glutathione (GSH), protein carbonyls, and malondialdehyde (MDA). Because MEO-only groups were not included and each chronic exposure condition was represented by a single treatment tank, the study addresses treatment-associated rescue/protection from SCO-related alterations rather than intrinsic MEO effects or independently replicated tank-level exposure effects.

## 2. Results

### 2.1. Gas Chromatography–Mass Spectrometry (GC-MS) Analysis of Mentha spicata L. Essential Oil

GC-MS/GC-FID profiling of MEO obtained from aerial parts collected in Elazig identified 12 reported constituents that together account for approximately 93.4% of the summed relative peak area, as shown in Table 1. The preparation is therefore described conservatively as carvone-rich, with carvone (67.8%), limonene (10.6%), and 1,8-cineole (4.2%) as the most abundant reported constituents. The remaining approximately 6.6% is not represented among these 12 tabulated constituents and cannot be assigned from the information available in the present dataset.

Carvone was the predominant reported constituent (67.8%), followed by limonene (10.6%), 1,8-cineole (4.2%), trans-dihydrocarvone (2.1%), β-myrcene (1.8%), cis-carvone oxide (1.5%), carveol (1.4%), cis-dihydrocarvone (1.2%), β-caryophyllene (1.0%), germacrene D (0.7%), α-pinene (0.6%), and bicyclogermacrene (0.5%). This compositional profile supports the descriptor “carvone-rich” but does not identify the constituent or combination of constituents responsible for the biological responses to the whole oil.

The single chromatographic profile should not be used to infer essential-oil “quality”, genetic or ecological stability, or post-harvest transformations. In addition, the HP-5MS procedure used here is non-chiral; therefore, the enantiomeric composition of carvone was not determined, and the present whole-oil findings are not assigned specifically to S-(+)- or R-(−)-carvone.

The reported composition is compatible with carvone/limonene-rich *M. spicata* profiles described in the literature, but the administered material was the complete essential oil. Contributions from limonene, 1,8-cineole, minor constituents, and additive, synergistic, or antagonistic interactions remain possible.

### 2.2. Behavioral Results

#### 2.2.1. MEO-Associated Attenuation of SCO-Related Anxiety-like Behavior (NTT)

**Figure 1:**
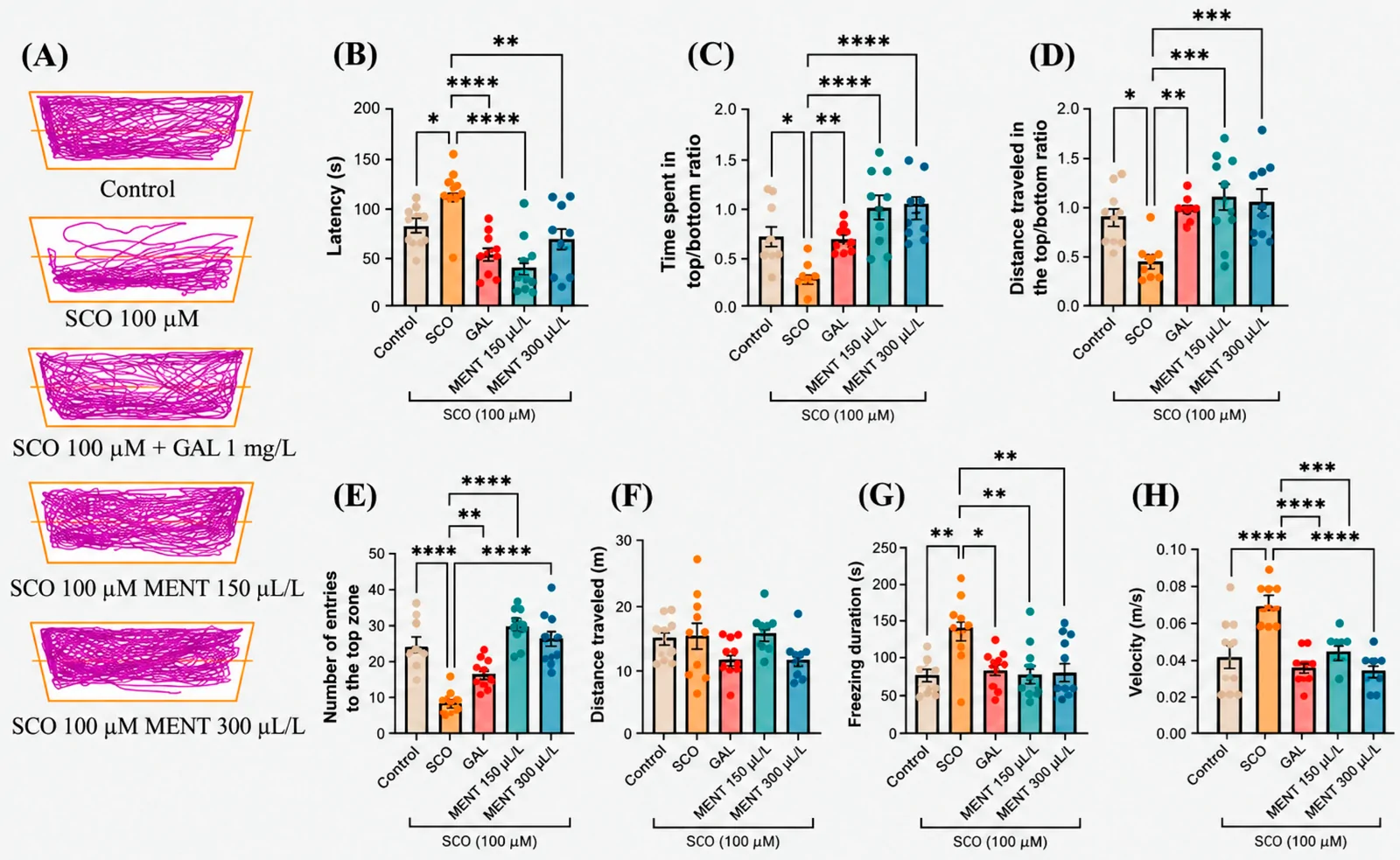
Effects of *Mentha spicata* L. essential oil (MEO) on scopolamine hydrobromide trihydrate (SCO)-induced anxiety-like behavior in zebrafish assessed by the novel tank diving test (NTT). (**A**) Representative swimming trajectories for each group: Control, SCO (100 μM), GAL (1 mg/L + SCO), and MEO (150 and 300 μL/L). SCO induced an anxiogenic phenotype characterized by predominant dwelling in the lower zone, whereas GAL and MEO restored exploratory activity. (**B**) Latency to first entry into the upper zone (s). (**C**) Time spent in the upper zone (s). (**D**) Distance ratio between upper and lower zones. (**E**) Number of entries into the upper zone. (**F**) Duration of immobility (s). (**G**) Total distance traveled (m). (**H**) Average swimming speed (m/s). Data are presented as mean ± SEM (n = 10/group). Statistical analyses were performed using one-way ANOVA followed by Tukey’s post hoc test. * *p* < 0.05, ** *p* < 0.01, *** *p* < 0.001, **** *p* < 0.0001 vs. SCO group. Study-wide behavioral endpoint multiplicity was additionally addressed by Benjamini–Hochberg FDR correction.

Representative swimming trajectories illustrated distinct behavioral profiles across experimental groups (Figure 1A). Control zebrafish displayed balanced vertical exploration, alternating between the upper and lower zones of the tank. In contrast, SCO exposure (100 μM) induced a pronounced anxiogenic phenotype, characterized by predominant dwelling in the lower zone, delayed transitions to the upper zone, prolonged immobility, and increased swimming speed. GAL treatment (1 mg/L) partially restored exploratory activity, whereas chronic MEO exposure at both concentrations (150 and 300 μL/L) produced robust anxiolytic effects, comparable to or exceeding those of GAL.

In the NTT (Figure 1), treatment significantly affected six of the seven prespecified endpoints. Omnibus statistics were: latency to the upper zone, F(4, 45) = 12.08, *p* = 9.46 × 10^−7^, q = 2.43 × 10^−6^, R^2^ = 0.5177 (Figure 1B); top/bottom time ratio, F(4, 45) = 10.88, *p* = 2.96 × 10^−6^, q = 6.66 × 10^−6^, R^2^ = 0.4916 (Figure 1C); top/bottom distance ratio, F(4, 45) = 7.30, *p* = 0.000129, q = 0.000193, R^2^ = 0.3934 (Figure 1D); upper-zone entries, F(4, 45) = 26.29, *p* = 2.81 × 10^−11^, q = 5.05 × 10^−10^, R^2^ = 0.7003 (Figure 1E); freezing duration, F(4, 45) = 5.46, *p* = 0.00113, q = 0.00136, R^2^ = 0.3269 (Figure 1G); and velocity, F(4, 45) = 14.31, *p* = 1.31 × 10^−7^, q = 3.92 × 10^−7^, R^2^ = 0.5598 (Figure 1H). Total distance did not differ significantly among groups [F(4, 45) = 2.15, *p* = 0.08996, q = 0.08996, R^2^ = 0.1605 (Figure 1F)]. SCO increased latency and bottom-oriented behavior and reduced upper-zone entries, whereas GAL and both MEO + SCO groups attenuated several SCO-associated changes. These findings are described as treatment-associated attenuation of SCO-related anxiety-like behavior rather than intrinsic anxiolytic activity because MEO-only controls were not included. Exact omnibus statistics, 95% CIs for R^2^, and complete Tukey-adjusted comparisons are provided in Supplementary Data S1 (Omnibus_stats and Tukey_posthoc).

#### 2.2.2. MEO-Associated Effects on Exploratory and Anxiety-like Behavior (NAT)

**Figure 2:**
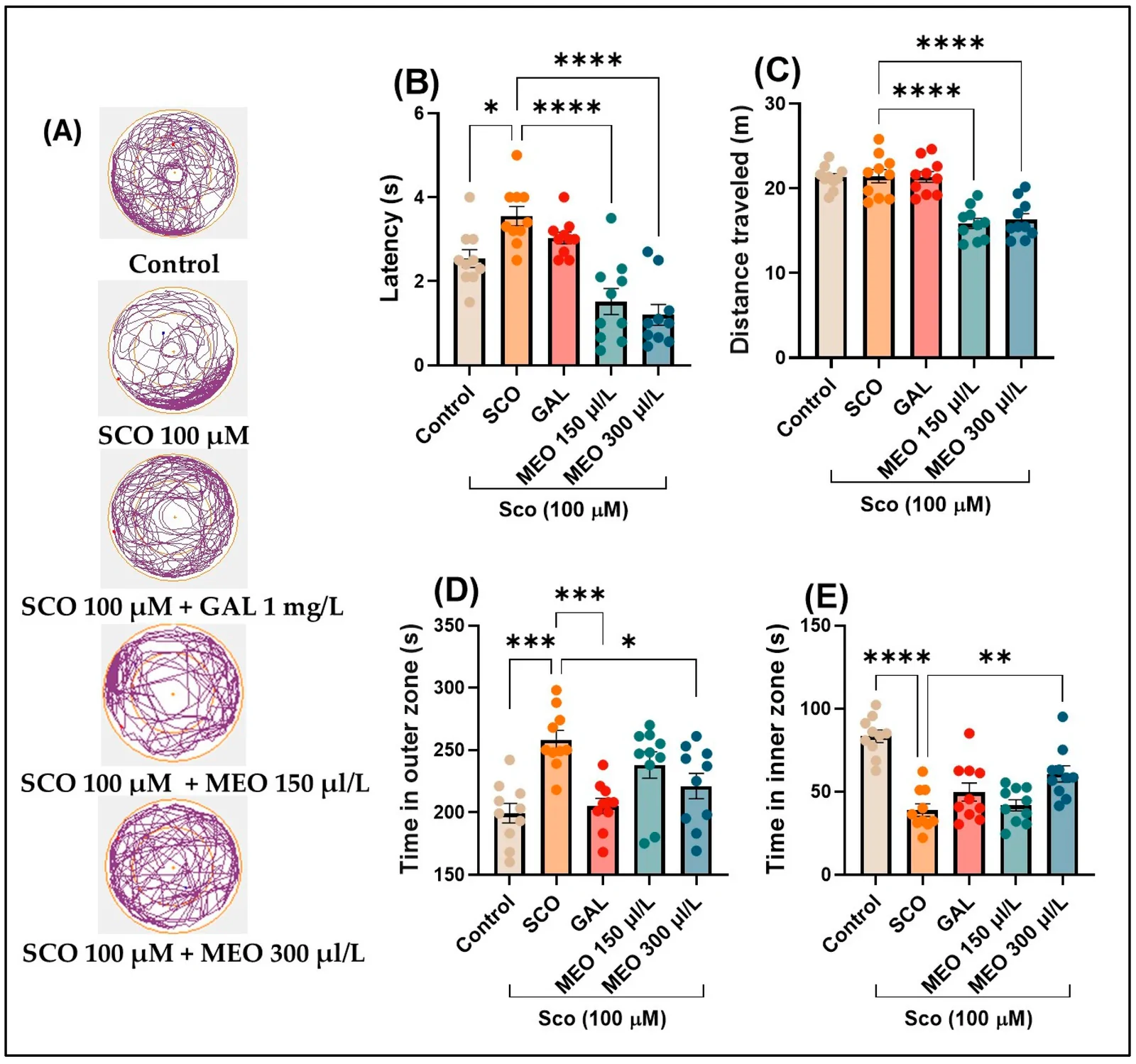
Effects of MEO on anxiety-like behavior in the novel approach test (NAT). (**A**) Representative trajectories for each group: Control, SCO (100 μM), GAL (1 mg/L + SCO), and MEO (150 and 300 μL/L). SCO induced pronounced thigmotaxis, whereas GAL and MEO promoted exploration of the center. (**B**) Latency to first entry into the central zone (s). (**C**) Total distance traveled (m). (**D**) Time spent in the peripheral zone (s). (**E**) Time spent in the central zone (s). Data are presented as mean ± SEM (n = 10/group). Statistical analyses were performed using one-way ANOVA followed by Tukey’s post hoc test. * *p* < 0.05, ** *p* < 0.01, *** *p* < 0.001, **** *p* < 0.0001 vs. SCO group. Study-wide behavioral endpoint multiplicity was additionally addressed by Benjamini–Hochberg FDR correction.

Swimming trajectory plots (Figure 2A) revealed clear group differences. SCO-exposed fish showed pronounced avoidance of the central zone, spending more time near the tank walls (thigmotaxis), a hallmark of anxiety-like behavior. In contrast, treatment with GAL and chronic MEO administration (150 and 300 μL/L) promoted active exploration of the center, indicating reduced anxiety.

In the NAT (Figure 2), treatment significantly affected all four prespecified endpoints: latency to the inner zone, F(4, 45) = 18.07, *p* = 6.47 × 10^−9^, q = 2.91 × 10^−8^, R^2^ = 0.6163 (Figure 2B); total distance, F(4, 45) = 19.88, *p* = 1.74 × 10^−9^, q = 1.04 × 10^−8^, R^2^ = 0.6387 (Figure 2C); outer-zone time, F(4, 45) = 7.89, *p* = 0.0000661, q = 0.000108, R^2^ = 0.4122 (Figure 2D); and inner-zone time, F(4, 45) = 17.19, *p* = 1.27 × 10^−8^, q = 4.56 × 10^−8^, R^2^ = 0.6044 (Figure 2E).

MEO + SCO groups attenuated several SCO-associated thigmotaxis-related endpoints. However, total distance was lower in the MEO groups even though Control, SCO, and GAL showed comparable values; therefore, the locomotor reduction cannot be interpreted as correction of SCO-induced hyperactivity and may include a direct MEO-related hypoactive component. This uncertainty is especially relevant at 300 µL/L and cannot be resolved without MEO-only controls. Exact omnibus statistics, 95% CIs for R^2^, and complete Tukey-adjusted comparisons are provided in Supplementary Data S1.

#### 2.2.3. MEO-Associated Effects on Y-Maze Performance

**Figure 3:**
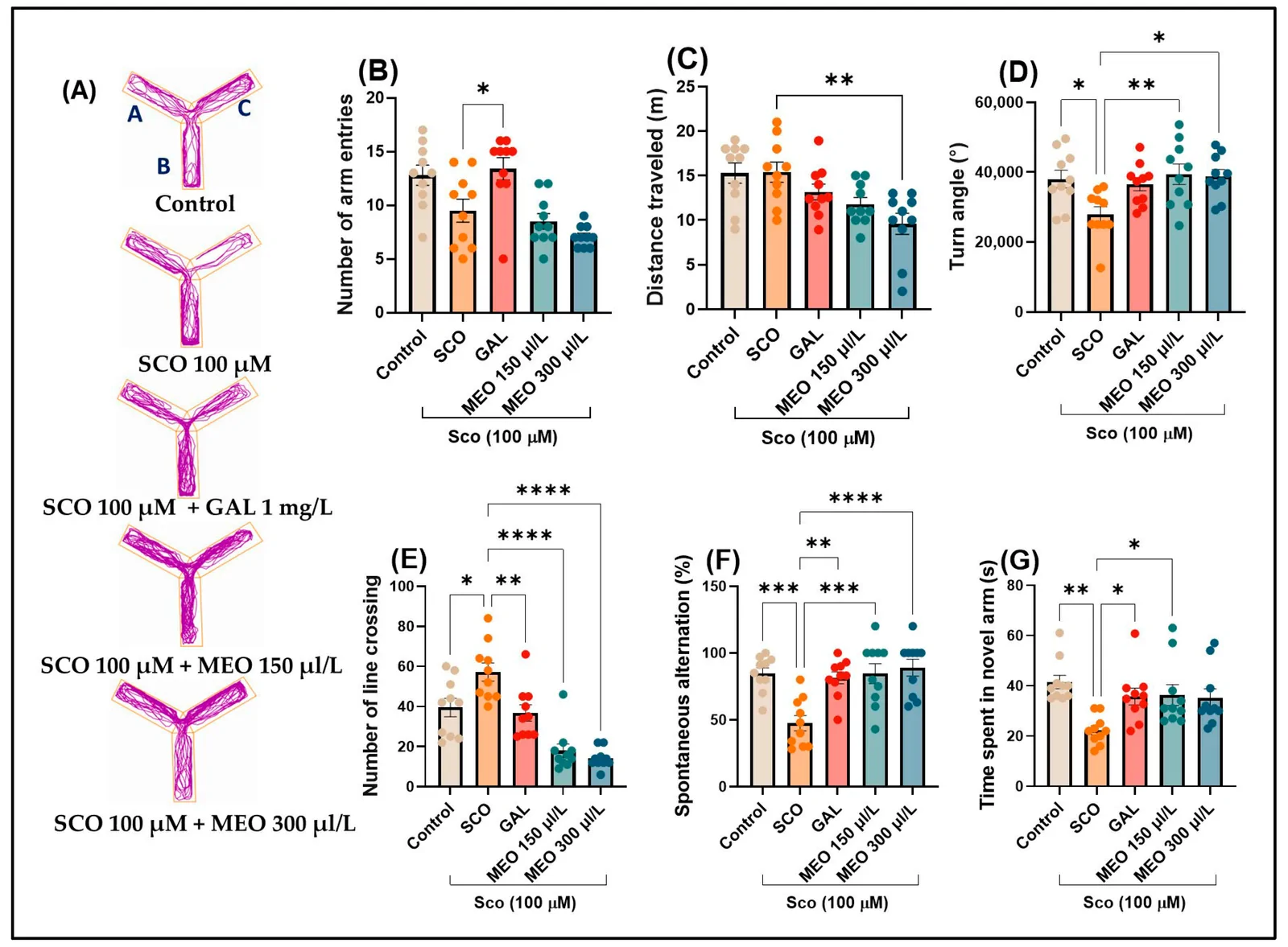
Effects of *Mentha spicata* L. essential oil (MEO) in the Y-shaped maze (Y-maze (**A**–**G**)). (**A**) Representative trajectories (track plots) for Control, scopolamine hydrobromide trihydrate (SCO, 100 μM), galantamine (GAL, 1 mg/L) + SCO, and MEO (150 and 300 μL/L) + SCO. (**B**) Number of arm entries; (**C**) distance traveled (m); (**D**) turn angle (°); (**E**) number of line crossings; (**F**) spontaneous alternations (%); (**G**) time spent in the novel arm (s). Data are presented as mean ± standard error of the mean (SEM; n = 10/group). Statistical analysis: one-way analysis of variance (ANOVA) followed by Tukey’s post hoc test. Significance: * *p* < 0.05, ** *p* < 0.01, *** *p* < 0.001, **** *p* < 0.0001. Study-wide behavioral endpoint multiplicity was additionally addressed by Benjamini–Hochberg FDR correction.

Trajectory plots (Figure 3A) revealed clear differences between groups. SCO exposure disrupted exploratory behavior, while GAL and MEO treatments distinctly modulated both locomotor and cognitive parameters.

In the Y-maze (Figure 3), treatment significantly affected arm entries [F(4, 45) = 10.04, *p* = 6.80 × 10^−6^, q = 1.36 × 10^−5^, R^2^ = 0.4715] (Figure 3B), total distance [F(4, 45) = 5.65, *p* = 0.000898, q = 0.00116, R^2^ = 0.3344] (Figure 3C), turn angle [F(4, 45) = 4.11, *p* = 0.00636, q = 0.00673, R^2^ = 0.2676] (Figure 3D), line crossings [F(4, 45) = 21.68, *p* = 5.07 × 10^−10^, q = 4.56 × 10^−9^, R^2^ = 0.6583] (Figure 3E), spontaneous alternation [F(4, 45) = 8.62, *p* = 0.0000297, q = 0.0000534, R^2^ = 0.4339] (Figure 3F), and novel-arm time [F(4, 45) = 4.87, *p* = 0.00240, q = 0.00270, R^2^ = 0.3019] (Figure 3G). SCO altered spatial-memory-related and exploratory outcomes. GAL and MEO 150 µL/L showed the clearest attenuation of the SCO-associated reduction in novel-arm exploration, whereas both MEO concentrations reduced several locomotor/exploratory indices. The lower concentration therefore provided the clearer memory-related signal with less evidence of locomotor interference; interpretation of the 300 µL/L group remains sensitive to hypoactivity. Exact omnibus statistics, 95% CIs for R^2^, and complete Tukey-adjusted comparisons are provided in Supplementary Data S1.

#### 2.2.4. MEO-Associated Effects on Novel Object Recognition (NOR)

**Figure 4:**
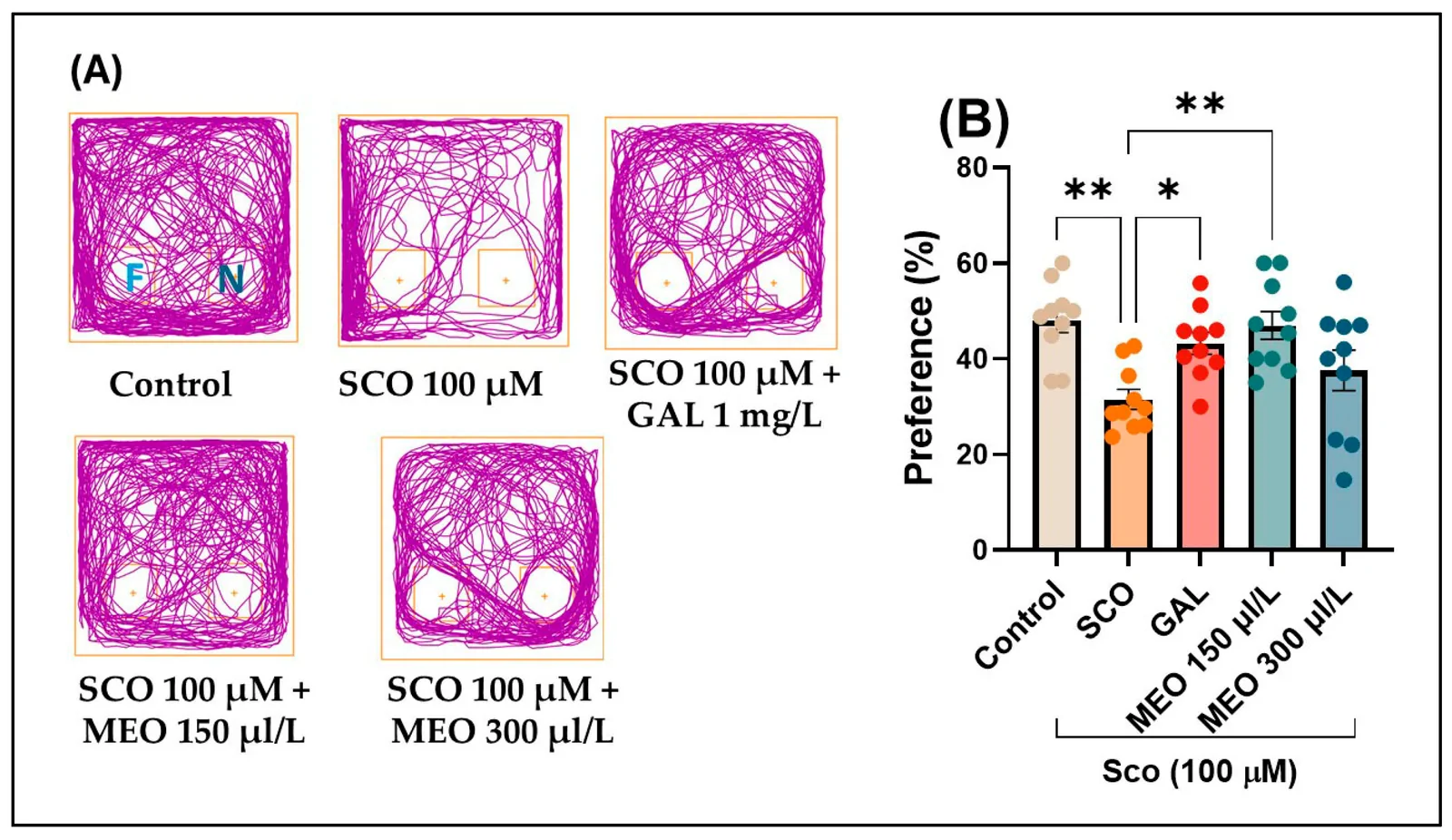
Novel object recognition test (NOR). (**A**) Representative trajectories during the test phase (familiar object, F; novel object, N) for Control, Scopolamine hydrobromide trihydrate (SCO, 100 μM), galantamine (GAL, 1 mg/L) + SCO, and *Mentha spicata* L. essential oil (MEO, 150 and 300 μL/L) + SCO groups. (**B**) Preference index (%) for NO in Control, SCO, GAL + SCO, MEO 150 μL/L, and MEO 300 μL/L. Data are presented as mean ± SEM (n = 10/group). Statistical analysis: one-way ANOVA followed by Tukey’s post hoc test. Significance levels: * *p* < 0.05, ** *p* < 0.01, (post hoc comparisons). Study-wide behavioral endpoint multiplicity was additionally addressed by Benjamini–Hochberg FDR correction.

Representative trajectories (Figure 4A) were recorded with an overhead camera and analyzed using ANY-maze v7.48. The paths (violet) show each fish’s movement during the test phase, with the positions of the familiar (F) and novel (N) objects marked. SCO exposure was characterized by predominant thigmotaxis (wall-following) and few visits near N, whereas Control, GAL, and MEO 150 μL/L displayed repeated entries into the N zone and more balanced arena exploration. MEO 300 μL/L showed an intermediate profile, with interactions around N present but less frequent than in GAL and MEO 150 μL/L groups.

NOR preference differed significantly among groups (Figure 4): F(4, 45) = 5.65, *p* = 0.000904, q = 0.00116, R^2^ = 0.3342 (Figure 4B). SCO reduced novel-object preference relative to Control, whereas GAL and MEO 150 µL/L attenuated this SCO-associated deficit; the 300 µL/L group showed a weaker memory-related response. The result supports a treatment-associated effect within the SCO challenge paradigm but does not establish an intrinsic procognitive action of MEO. Exact omnibus statistics, the 95% CI for R^2^, and complete Tukey-adjusted comparisons are provided in Supplementary Data S1.

### 2.3. Biochemical Results

AChE activity, antioxidant enzymes (SOD, CAT, and GPX), GSH, protein carbonyls, and MDA were assessed in three independent individual zebrafish brains per group (n = 3/group), randomly selected from the behaviorally tested cohort; tissues were not pooled. Each biological sample was assayed in duplicate, and duplicate analytical readings were averaged before statistical analysis and were not treated as independent observations. Because of the limited biological n, these biochemical results are considered supportive and require confirmation in larger independently replicated cohorts. Raw individual biochemical values are provided in Supplementary Data S1 (Biochemistry_raw); exact omnibus *p*-values, FDR-adjusted q values, R^2^ effect sizes with 95% CIs, permutation sensitivity analyses, and complete post hoc matrices are provided in Omnibus_stats and Tukey_posthoc, while model diagnostic plots are provided in Supplementary Figure S1.

**Figure 5:**
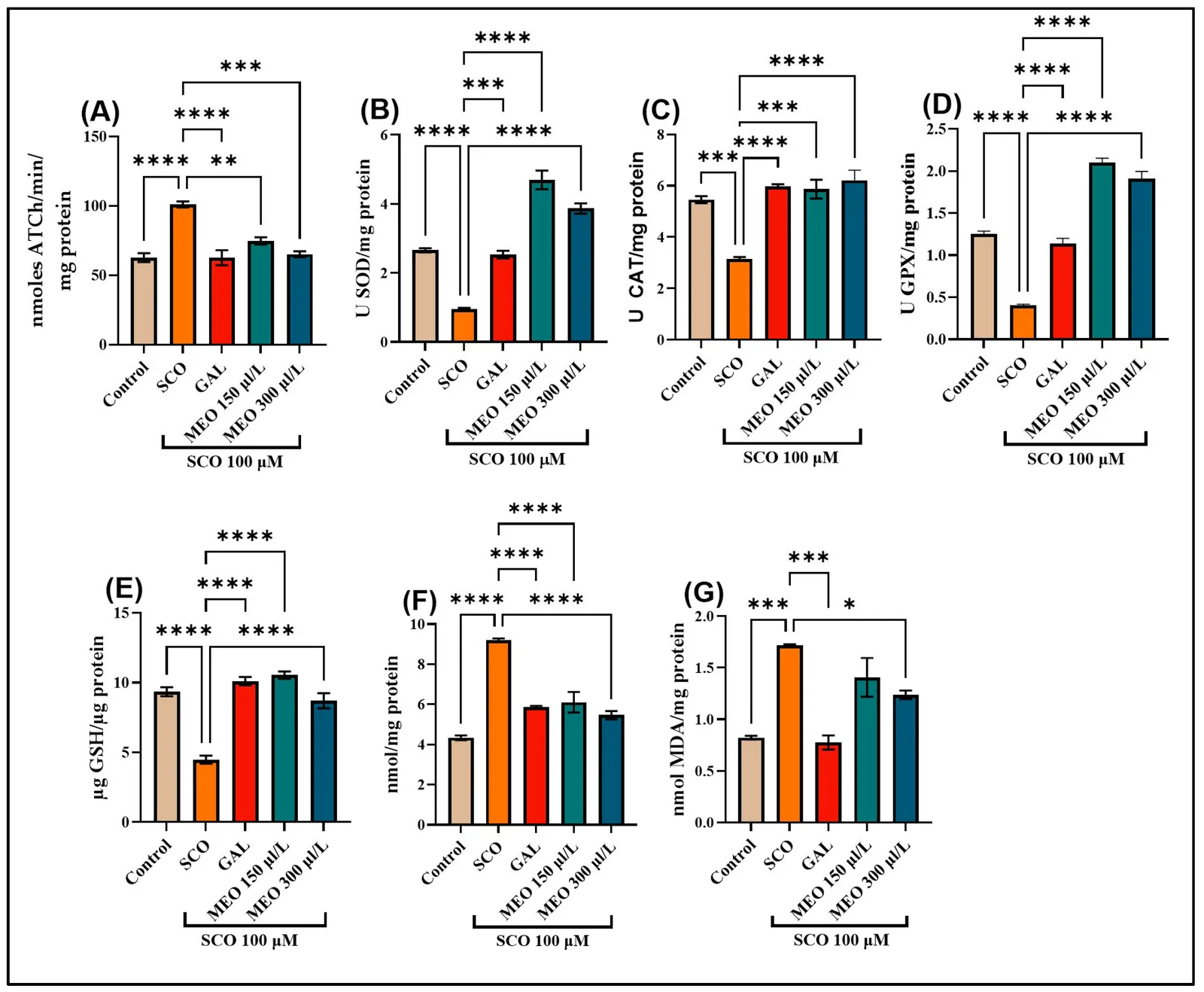
Effects of SCO (scopolamine hydrobromide trihydrate, 100 µM), GAL (galantamine, 1 mg/L), and MEO (*Mentha spicata* L. essential oil, 150 or 300 µL/L) on: (**A**) AChE activity (acetylcholinesterase; nmol/mg protein); (**B**) SOD (superoxide dismutase; specific activity, U/mg protein); (**C**) CAT (catalase; µmol H_2_O_2_/min/mg protein); (**D**) GPX (glutathione peroxidase; U/mg protein); (**E**) GSH (reduced glutathione; µmol/mg protein); (**F**) protein carbonyls (nmol DNPH/mg protein; DNPH = 2,4-dinitrophenylhydrazine); (**G**) MDA (malondialdehyde; nmol/mg protein). Bars represent mean ± SEM, with individual values shown (n = 3/group; duplicate determinations). Statistical analysis: one-way ANOVA followed by Tukey’s post hoc test; homogeneity of variances verified by the Brown–Forsythe test (all *p* > 0.05). Significance levels: * *p* < 0.05, ** *p* < 0.01, *** *p* < 0.001, **** *p* < 0.0001. Study-wide behavioral endpoint multiplicity was additionally addressed by Benjamini–Hochberg FDR correction. Complete pairwise comparisons, exact statistics, effect-size estimates, and raw individual values are provided in Supplementary Data S1. The corresponding raw values are available in Supplementary Data S1 (sheet Biochemistry_raw), with omnibus statistics and complete Tukey-adjusted comparisons in sheets Omnibus_stats and Tukey_posthoc.

Treatment significantly affected all seven biochemical endpoints (Figure 5): AChE, F(4, 10) = 23.97, *p* = 0.0000416, q = 0.0000485, R^2^ = 0.9055 (Figure 5A); SOD, F(4, 10) = 89.97, *p* = 8.47 × 10^−8^, q = 2.97 × 10^−7^, R^2^ = 0.9730 (Figure 5B); CAT, F(4, 10) = 24.47, *p* = 0.0000379, q = 0.0000485, R^2^ = 0.9073 (Figure 5C); GPX, F(4, 10) = 155.76, *p* = 5.82 × 10^−9^, q = 4.08 × 10^−8^, R^2^ = 0.9842 (Figure 5D); GSH, F(4, 10) = 46.39, *p* = 2.01 × 10^−6^, q = 3.51 × 10^−6^, R^2^ = 0.9489 (Figure 5E); protein carbonyls, F(4, 10) = 50.40, *p* = 1.36 × 10^−6^, q = 3.17 × 10^−6^, R^2^ = 0.9527 (Figure 5F); and MDA, F(4, 10) = 18.86, *p* = 0.000119, q = 0.000119, R^2^ = 0.8829 (Figure 5G). Permutation-based sensitivity tests also supported a treatment effect for all seven endpoints (exact permutation *p*-values in Supplementary Data S1). Relative to SCO, both MEO concentrations reduced AChE activity, increased SOD, CAT, GPX, and GSH, and reduced protein carbonyls. For MDA, 300 µL/L significantly reduced the SCO-associated elevation, whereas 150 µL/L did not. Because n = 3 biological brains/group, these findings are interpreted as supportive rather than definitive. Raw values, exact omnibus *p*-values and q values, 95% CIs for R^2^, permutation results, and complete Tukey-adjusted matrices are provided in Supplementary Data S1 (Biochemistry_raw, Omnibus_stats, and Tukey_posthoc); residual-versus-fitted and Q–Q plots are provided in Supplementary Figure S1.

### 2.4. Exploratory Treatment-Adjusted Behavior–Biomarker Associations

**Figure 6:**
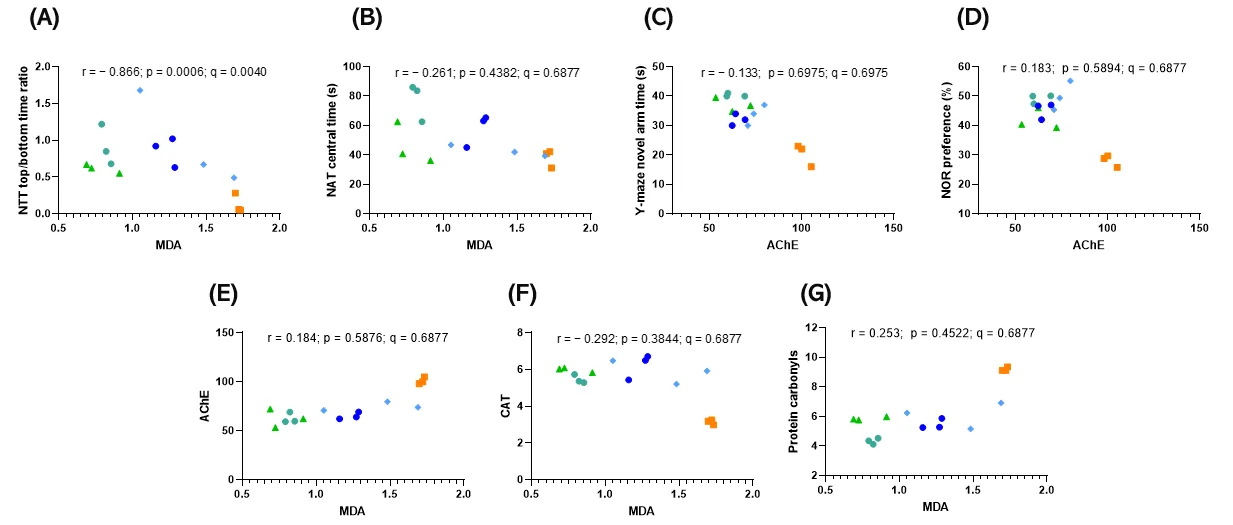
Exploratory behavior–biomarker associations using the 15 paired fish for which biochemical and behavioral data were both available. Points are coded by treatment group. Panels show: (**A**) MDA vs. NTT top/bottom time ratio; (**B**) MDA vs. NAT central-zone time; (**C**) AChE vs. Y-maze novel-arm time; (**D**) AChE vs. NOR preference; (**E**) MDA vs. AChE; (**F**) MDA vs. CAT; and (**G**) MDA vs. protein carbonyls. Displayed r, *p*-, and q values are from treatment-adjusted analyses and Benjamini–Hochberg FDR correction. Only panel (**A**) remained significant after adjustment. These associations are exploratory and do not imply causality.

Each biochemical value was matched to the corresponding behavioral value obtained from the same fish, yielding three paired observations per group (n = 15). Because pooling treatment groups can generate ecological or treatment-clustered correlations, all seven associations shown in Figure 6 were reanalyzed using linear regression with treatment group included as a categorical covariate, followed by Benjamini–Hochberg correction across the seven tests. Only the inverse association between MDA and the NTT top/bottom time ratio remained statistically significant after adjustment (Figure 6A; partial r = −0.866, *p* = 0.000567, q = 0.00397).

In contrast, the treatment-adjusted associations between MDA and NAT central-zone time (Figure 6B), AChE activity and Y-maze novel-arm time (Figure 6C), AChE activity and NOR preference (Figure 6D), AChE activity and MDA (Figure 6E), CAT activity and MDA (Figure 6F), and protein carbonyls and MDA (Figure 6G) were not statistically significant (*p* = 0.384–0.698; q ≥ 0.688). These findings indicate that the apparently strong crude Pearson correlations for these six relationships were largely driven by between-group treatment differences rather than by consistent within-group associations. A residualized Spearman sensitivity analysis further supported the MDA–NTT relationship (Figure 6A; ρ = −0.743, *p* = 0.00151), whereas none of the remaining associations were supported by this analysis. Accordingly, the correlation analysis should be regarded as exploratory and associative and does not establish causality or demonstrate a specific cholinergic–redox mechanism.

### 2.5. In Silico ADMET Profiling of the Major Constituents of Mentha spicata Essential Oil

After the experimental behavioral, biochemical, and correlational findings were established, computational descriptors were examined for selected MEO constituents as an exploratory plausibility analysis. All values in this section are model-derived outputs rather than measured pharmacokinetic endpoints. SwissADME calculated low molecular weights (136.23–154.25 g/mol), very low polarity (TPSA 0.00–17.07 Å^2^), and limited hydrogen-bonding capacity (0 HBD; 0–1 HBA) for the major volatile markers (Table 2). These descriptors are relevant to passive permeability, but no single descriptor establishes membrane or BBB penetration. Experimental BBB studies show that lipophilicity must be interpreted together with molecular size, polarity, ionization, and other disposition factors. The in silico component was substantially reduced and is presented only as exploratory physicochemical context [17].

**Figure 7:**
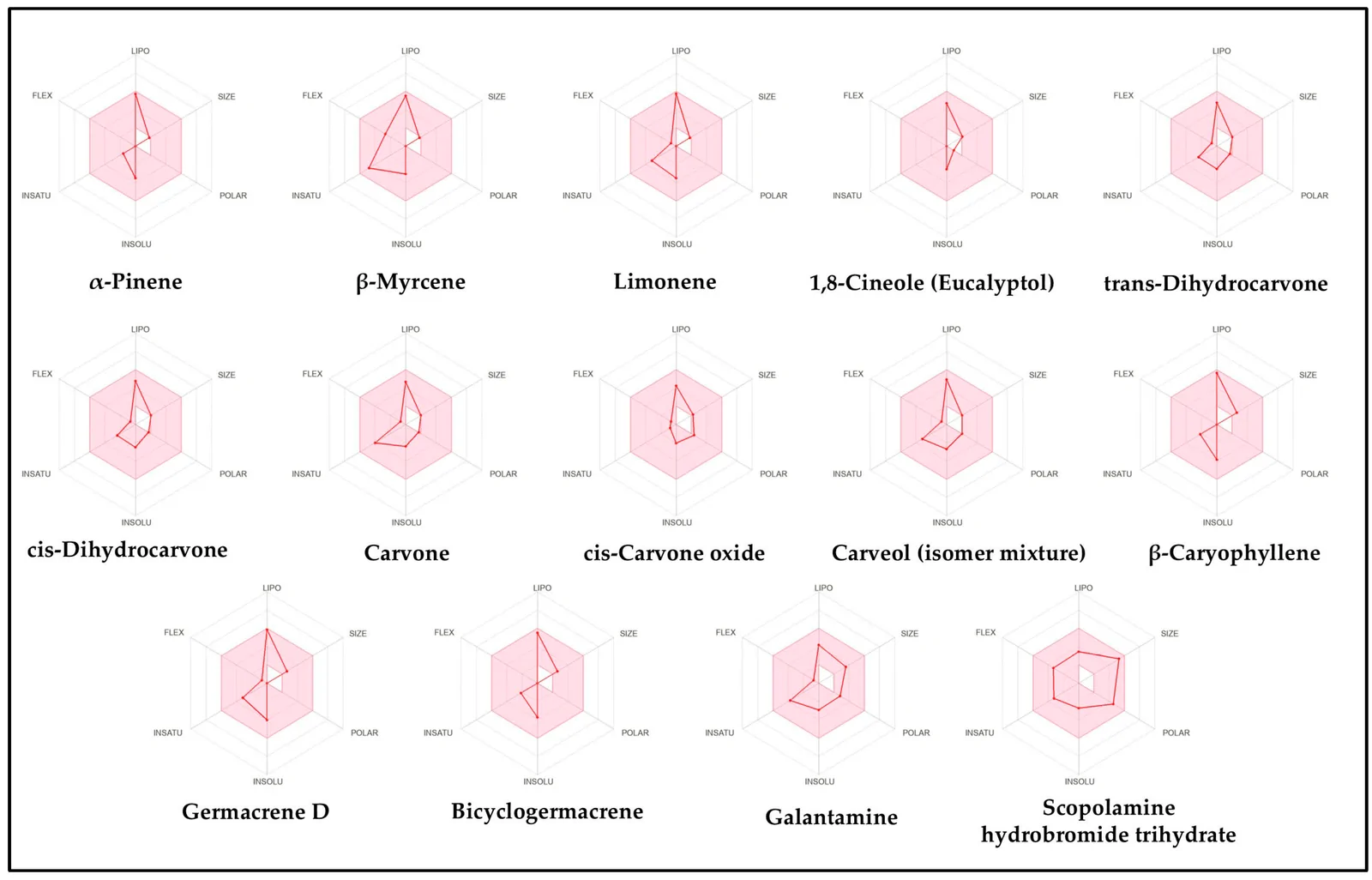
SwissADME bioavailability-radar plots for the evaluated compounds. The pink region denotes the platform’s preferred physicochemical ranges for six descriptors: lipophilicity, molecular size, polarity, solubility, saturation, and flexibility. These plots are computational descriptors and do not constitute experimental evidence of oral absorption, bioavailability, membrane permeability, BBB transport, or CNS exposure.

Figure 7 summarizes the SwissADME bioavailability-radar descriptors. The pink area represents the platform’s preferred physicochemical ranges and should not be interpreted as measured oral absorption or bioavailability. Because several monoterpenes are very small and weakly polar, the radar is used here only for comparative physicochemical description rather than to claim a favorable pharmacokinetic profile.

All five selected constituents met the Lipinski and Veber filters (0 violations) (Table 3), whereas the Ghose and Muegge filters flagged size-related constraints for these small monoterpenes. These rule-based filters are heuristic drug-likeness descriptors and were not interpreted as proof of oral bioavailability. GAL and SCO are included as reference compounds only; direct cross-comparison should be interpreted cautiously because simple physicochemical filters do not reproduce the full pharmacology of ionized compounds or salt forms.

#### 2.5.1. Absorption and Permeability

ADMET-AI [18] assigned high model probabilities for human intestinal absorption and oral bioavailability to the selected essential-oil markers (Table 4). These numbers are computational probabilities rather than measured absorption data and are not directly transferable to the zebrafish immersion route used in this study.

ADMET-AI also generated PAMPA and cell-permeability estimates for the monoterpenes and reference compounds (Table 4). We report these values descriptively. Their apparent favorability for several monoterpenes does not demonstrate uptake in zebrafish or humans because passive permeability is influenced by lipophilicity, ionization, protein binding, transporters, and experimental context [16]. The lower model scores for SCO are therefore not interpreted as evidence of limited CNS access.

#### 2.5.2. Distribution and BBB/CNS Relevance

**Figure 8:**
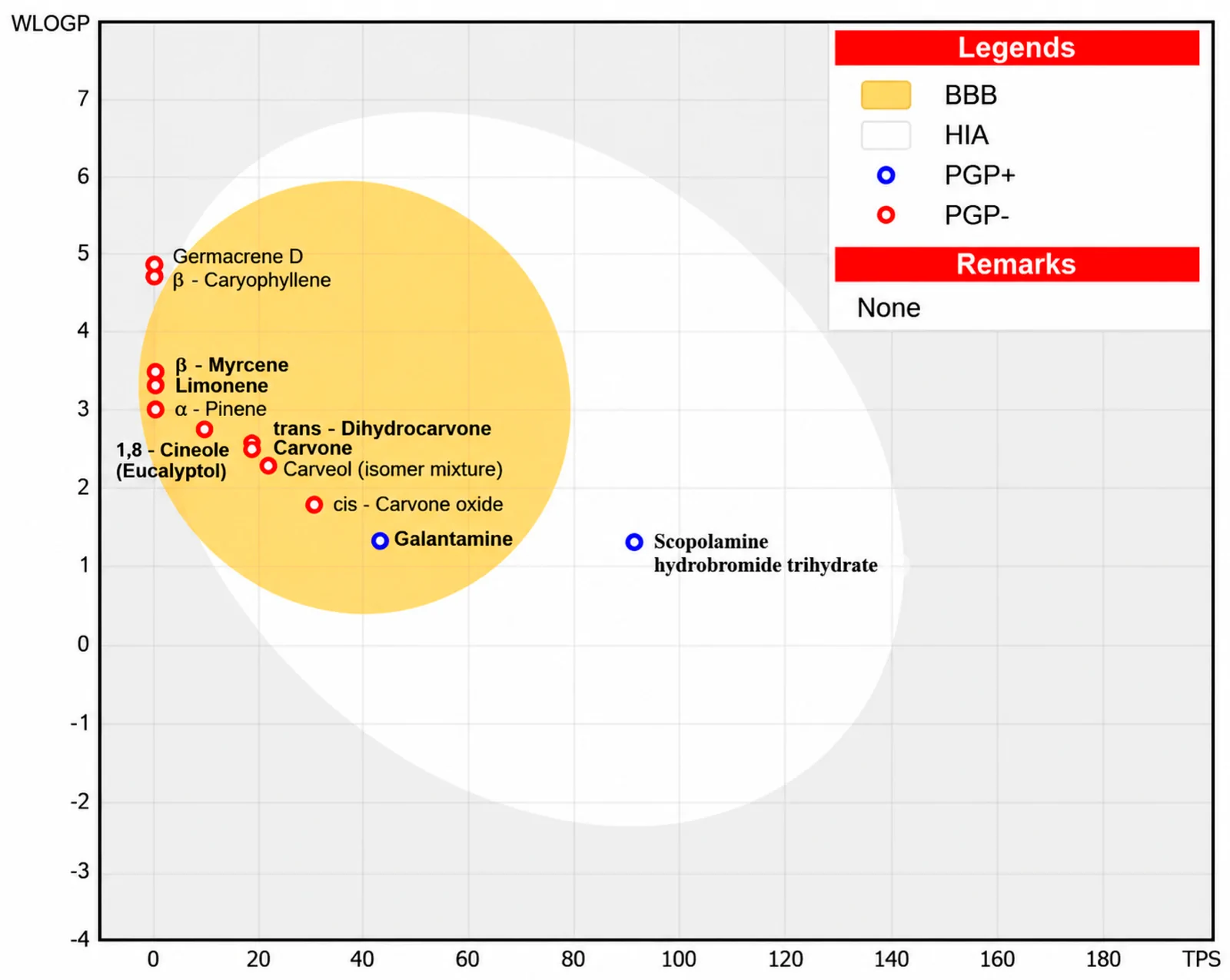
SwissADME BOILED-Egg plot for the reference compounds and selected major constituents of *Mentha spicata* L. essential oil (MEO). The model maps WLOGP against TPSA to provide a qualitative prediction of passive gastrointestinal absorption and BBB permeability and annotates predicted P-glycoprotein status. It is a computational classification and should not be interpreted as a direct measurement of BBB transport, brain concentration, or CNS retention.

Figure 8 displays the SwissADME BOILED-Egg classification, which combines WLOGP (lipophilicity) and TPSA (polarity). This two-parameter model is useful for visualizing physicochemical space associated with passive gastrointestinal and BBB permeability, but it is not a permeability assay. The major monoterpenes occupy a low-polarity and relatively lipophilic region of the plot, a combination compatible with passive membrane partitioning. However, experimental BBB studies demonstrate that lipophilicity acts together with molecular size, ionization, and other determinants; therefore, low TPSA or limited HBD/HBA alone is insufficient to infer brain exposure [16]. For S-(+)-carvone, independent experimental work has reported detectable hippocampal exposure [15], but no equivalent brain-level measurements were made in the present zebrafish study.

GAL and SCO are included as pharmacological reference compounds, but their predicted values are not used to validate the computational model. In particular, the comparatively low BBB score assigned to scopolamine hydrobromide trihydrate (0.35) conflicts with its established central pharmacology and with experimental LC-MS/MS data showing scopolamine in rat brain, including the hippocampus and cortex, after systemic administration [19]. This discrepancy illustrates a limitation of applying diffusion-based predictions to ionizable compounds and salt/hydrate representations. Accordingly, the SCO prediction is treated as a model limitation rather than as evidence of poor CNS uptake.

Table 5 therefore reports distribution-related values as computational outputs only. High model scores for the MEO markers indicate compatibility with the model’s BBB-permeable class, not demonstrated cerebral exposure. Conversely, the low SCO score should not be interpreted biologically because experimental brain distribution has been measured directly [19]. Direct tissue-level pharmacokinetic measurements would be required to establish whether the major MEO constituents reach the zebrafish brain under the present immersion protocol.

Plasma protein binding (PPB) was predicted to be highest for β-myrcene (91.14%) and limonene (83.97%), suggesting extensive binding/partitioning, whereas the oxygenated monoterpenes (carvone, trans-dihydrocarvone, and 1,8-cineole) showed moderate PPB (~56–61%). The steady-state volume of distribution (VDss) was greatest for β-myrcene (9.12 L/kg), intermediate for limonene (3.53 L/kg) and 1,8-cineole (1.47 L/kg), and within a comparable range to the reference compounds (GAL: 2.00 L/kg; SCO: 4.06 L/kg).

#### 2.5.3. Metabolism and Excretion

Across the volatile markers, predicted CYP inhibition probabilities were generally low, particularly for CYP2C9, CYP2D6, and CYP3A4 (Table 6). Notably, β-myrcene displayed comparatively high inhibition scores for CYP1A2 (0.32) and CYP2C19 (0.38), whereas GAL showed a markedly higher predicted CYP2D6 inhibition signal (0.54), consistent with a greater interaction potential typical of more complex CNS drugs.

ADMET-AI generated model-based half-life and clearance outputs (Table 7). The values of 0.00 returned for carvone and trans-dihydrocarvone were treated as unavailable or undefined model outputs and were not interpreted as biological half-lives. None of the values in Table 7 represent experimentally measured pharmacokinetics in the present study.

#### 2.5.4. Toxicity and Safety Flags

Toxicity screening suggested low predicted hERG blocking for carvone and trans-dihydrocarvone (0.01) and modest values for limonene/β-myrcene (0.10) and 1,8-cineole (0.20), whereas higher scores were observed for galantamine (0.61) and scopolamine hydrobromide trihydrate (0.49) (Table 8).

Mutagenicity signals were low for most monoterpenes (0.01–0.11) but higher for the reference compounds (GAL: 0.44; SCO: 0.37). β-myrcene showed the highest predicted skin reaction score (0.95) and the highest carcinogenicity score among the tested set (0.58), warranting cautious interpretation in the context of mixture exposure and route (immersion) and supporting conservative dosing windows. Finally, β-myrcene displayed the strongest NRF2/ARE-related signal (0.42), which may align mechanistically with the antioxidant profile observed experimentally, although this remains hypothesis-generating and requires experimental validation.

## 3. Discussion

The present study provides convergent chemical, behavioral, biochemical, and correlational evidence supporting MEO’s neuroactive and neuroprotective profile in a SCO-induced zebrafish model of cognitive impairment. Taken together, the data indicate that a carvone-dominant MEO chemotype attenuates SCO-induced anxiogenic and amnesic phenotypes while restoring cholinergic balance and antioxidant defenses in the zebrafish brain.

GC-MS/GC-FID showed a carvone-rich profile dominated by carvone (67.8%), limonene (10.6%), and 1,8-cineole (4.2%). This profile is compatible with carvone/limonene-rich M. spicata reports, but a single collection cannot support claims about oil quality, genetic/ecological stability, or post-harvest transformations. Because the chromatographic procedure was non-chiral, the carvone enantiomer present in the oil was not determined [13]. This compositional signature closely matches that reported for Anatolian/Turkish MEO, where carvone predominance is considered a hallmark of chemotype stability and oil quality. Such chemical consistency strengthens the biological interpretability of the observed effects and supports comparison with the existing neuropharmacological literature on spearmint-derived essential oils) [13,20].

SCO produced anxiety-like, exploratory, memory-related, cholinergic, and oxidative changes consistent with an acute pharmacological model of cholinergic hypofunction. This model is useful for mechanistic screening but should not be equated directly with human AD or progressive neurodegeneration [21], characterized by reduced vertical exploration and increased bottom-dwelling in the NTT, decreased center exploration and increased behavioral disorganization in the NAT, impaired spatial working memory in the Y-maze test, and reduced recognition memory in the NOR test. These behavioral alterations were accompanied by a pronounced pro-oxidant biochemical profile and increased AChE activity, fully consistent with the established zebrafish SCO model, which reproduces key features of cholinergic hypofunction and oxidative stress associated with AD-like pathology [3,9,21].

Within the SCO challenge paradigm, both MEO concentrations attenuated several anxiety-like behavioral endpoints. These effects are best described as anxiolytic-like treatment-associated changes rather than intrinsic anxiolytic activity. Reduced distance and/or entries at the higher concentration in selected tasks indicate that locomotor or arousal changes may influence exploration-dependent outcomes [21,22].

The interventions revealed two distinct patterns. GAL (positive control) restored most behavioral indices (Figure 1, Figure 2, Figure 3, Figure 4, Figure 5 and Figure 6) and normalized oxidative parameters (Figure 5), confirming that cholinergic improvement is closely linked to the reduction of oxidative stress. Our findings align with the previous literature. In a zebrafish model of chromium-induced neurotoxicity, GAL, alongside donepezil and resveratrol, was shown to protect against anxiety, memory deficits, and oxidative stress [23].

Memory-related performance improved most consistently at 150 µL/L, whereas 300 µL/L retained activity on selected behavioral and biochemical endpoints but showed greater evidence of reduced exploration. With only two MEO concentrations, these findings suggest a possible concentration-related pattern rather than a defined therapeutic window or a monotonic dose–response relationship.

This partial non-parallelism between cognitive endpoints is not uncommon in zebrafish essential-oil studies and likely reflects subtle shifts in arousal, motivation, or inhibitory tone rather than an absence of mnemonic benefit. Exploration-based tasks are particularly sensitive to such factors. Taken together, 150 µL/L appears to represent the clearest cognitive-response window, showing the most consistent improvement in memory-related endpoints with less evidence of locomotor interference. In contrast, 300 µL/L retained effects on anxiety-related and biochemical endpoints but was accompanied by reduced exploratory activity in selected tasks, consistent with a mild hypoactive component that may influence exploration-dependent readouts. Thus, the dose pattern appears endpoint-dependent rather than following a simple monotonic dose–response relationship.

Biochemically, SCO induced a marked increase in AChE activity and oxidative stress, as evidenced by reduced antioxidant enzyme activities (SOD, CAT, and GPX), depletion of GSH, and increased lipid peroxidation (MDA) and protein oxidation (protein carbonyls). This biochemical signature closely reproduces the mechanistic core of the SCO model and reinforces the validity of the present dataset.

Both MEO concentrations attenuated SCO-associated AChE hyperactivity and several redox abnormalities at the group level. However, the treatment-adjusted behavior–biomarker analysis substantially weakened the earlier mechanistic interpretation: only the inverse MDA-NTT top/bottom association remained significant after accounting for treatment group and FDR correction. The remaining crude correlations were largely influenced by between-group separation and should not be used as evidence of an individual-level cholinergic–redox mechanism.

MEO also robustly restored antioxidant defenses. SOD, CAT, and GPX activities were normalized or even upregulated above control levels, while protein carbonyls were markedly reduced, with near-complete normalization at the higher dose. Lipid peroxidation was partially reduced, more prominently at 300 μL/L, whereas the lower dose showed a stronger enzymatic antioxidant response with a more modest effect on MDA. This dissociation suggests that MEO may rapidly enhance enzymatic antioxidant capacity, while mitigation of membrane lipid damage may require higher exposure, longer treatment duration, or a reduced oxidative burden.

The biochemical analyses were based on three independent biological replicates per group, with duplicate technical determinations averaged within each biological sample. This limited biological replication reduces the precision and robustness of the estimates; accordingly, the biochemical findings should be regarded as supportive rather than definitive. To provide magnitude information independent of *p*-values, the one-way ANOVA R^2^ values for the seven biochemical endpoints were reported and ranged from 0.8829 (MDA) to 0.9842 (GPX), indicating large treatment-associated effects within this dataset. Importantly, these large observed effect sizes do not overcome the limitations imposed by the small biological sample and require confirmation in larger, independently replicated cohorts.

The exploratory correlation analysis was also directionally consistent with the redox findings: higher MDA was associated with more anxiety-like behavioral indices, CAT was inversely associated with MDA, and protein carbonyls were positively associated with MDA. These relationships remained significant after Benjamini–Hochberg correction but are interpreted as associative signals rather than evidence of a mechanistic coupling at the individual-animal level.

Because the correlation dataset comprised only 15 paired observations (three biological replicates from each of five treatment groups), the associations were treated as exploratory. Benjamini–Hochberg false-discovery-rate correction was applied across the seven reported correlations; all remained significant at q < 0.05. However, treatment-group separation may contribute to the observed relationships, and these correlations should not be interpreted as individual-level causal or mechanistic proof. The observed biochemical effects are chemically plausible in the context of the carvone-dominant composition of MEO, but the present study does not establish that carvone alone caused the neurobehavioral or biochemical effects. Carvone is the major constituent (67.8%), and independent literature reports support neuropharmacologically relevant properties, including cholinesterase-related activity, GABAA-receptor modulation, and experimentally demonstrated brain exposure for S-(+)-carvone [15,24,25]. These observations support carvone as a plausible contributor rather than a proven active principle. Because the whole essential oil was administered, the effects may also reflect additive, synergistic, or antagonistic interactions among carvone, limonene, 1,8-cineole, β-myrcene, and minor constituents. Constituent-specific dosing and reconstituted-mixture experiments will be required to distinguish the contribution of carvone from mixture effects. Secondary constituents further reinforce this mechanistic plausibility. 1,8-Cineole exhibits well-documented antioxidant and mitochondria-protective actions, including Nrf2 pathway activation and reductions in lipid and protein oxidation [26]. Limonene has been shown to modulate adenosinergic, dopaminergic, and GABAergic signaling, mechanisms relevant to anxiety regulation, arousal, and cognitive flexibility. Additionally, carvone enantiomers have been reported to modulate GABAA receptors in vitro, providing a plausible explanation for the anxiolytic-like phenotype and for the dose-dependent modulation of exploratory behavior observed in the present study [27,28].

Thus, beyond antioxidant effects alone, MEO likely acts through a multi-target neuromodulatory profile, integrating cholinergic, redox, and inhibitory/excitatory balance mechanisms. Such pleiotropy is characteristic of essential oils and may underlie their combined anxiolytic and procognitive actions.

Taken together, the composition of MEO and the external literature are compatible with a multi-target neuromodulatory profile involving cholinergic, redox, and inhibitory/excitatory pathways. However, the present whole-oil design cannot determine whether these effects arise from carvone, other constituents, or additive/synergistic interactions within the mixture; this mechanistic interpretation therefore remains hypothesis-generating.

The in silico analysis was used only after the experimental findings as a physicochemical plausibility check. BBB penetration cannot be inferred from low TPSA or limited hydrogen-bonding capacity alone; experimental studies show that lipophilicity, molecular size, ionization, membrane partitioning, protein binding, and transporter effects can jointly determine brain disposition [16]. Importantly, independent work has experimentally detected S-(+)-carvone in the hippocampus [15], whereas systemic scopolamine has been quantified by LC-MS/MS in rat hippocampus and cortex [19]. These empirical observations take precedence over the present model outputs. Thus, SwissADME and ADMET-AI predictions are hypothesis-generating and do not demonstrate zebrafish brain exposure, oral bioavailability, or pharmacokinetics of the essential-oil constituents.

Several limitations materially constrain interpretation. First, each treatment condition used a single 10 L exposure tank; thus, individual fish provide behavioral observations but are not independent chronic-exposure replicates, and retrospective tank-level reanalysis is not possible. Second, MEO-only groups were absent, preventing separation of rescue/protection from intrinsic MEO behavioral, locomotor, biochemical, or toxic effects. Third, the nominal MEO concentrations were not analytically verified in tank water, and no dedicated physicochemical stability study demonstrated stable exposure with 0.02% Tween 20. Fourth, biochemical analyses used three individual brains/group, limiting precision despite large observed effects. Fifth, the same cohort completed a fixed behavioral sequence, so residual habituation, learning, stress, or carry-over cannot be completely excluded despite recovery and cleaning procedures. Sixth, although sex was balanced at five males and five females/group, the study was not powered for sex-stratified inference. Finally, no direct brain or plasma quantification of MEO constituents was performed, and the SCO model represents acute pharmacological cholinergic disruption rather than chronic neurodegeneration.

Accordingly, the findings should be regarded as exploratory treatment-associated attenuation of SCO-related changes. Confirmation will require independently replicated treatment tanks, MEO-only controls, broader concentration testing, direct measurement of exposure in water and biological tissues, and adequately powered analyses of sex-related variability.

## 4. Materials and Methods

### 4.1. Plant Material and Essential Oil Preparation

Aerial parts of M. spicata were collected in June 2024 from Elazığ, Turkey. The plant material was authenticated by Prof. Dr. Eyup Bagci. A voucher specimen (No. 2024HFU) was deposited in the Herbarium of the Department of Biology, Fırat University, Elazığ, Turkey. The essential oil was obtained from the air-dried aerial parts by hydrodistillation. The yield was 0.7% (*v*/*w*), calculated relative to the dry mass of the plant material, and stored at 4 °C until chemical analysis and biological testing.

### 4.2. Gas Chromatography–Mass Spectrometry (GC-MS/GC-FID) Analysis

The essential oil was analyzed using an Agilent 6890 gas chromatograph coupled to an Agilent 5973N mass-selective detector and equipped with a flame-ionization detector (FID) (Agilent Technologies, Santa Clara, CA, USA), according to a previously reported method [29]. Separation was performed on an HP-5MS capillary column (30 m × 0.25 mm internal diameter; 0.25 μm film thickness). Helium was used as the carrier gas at a constant flow rate of 1 mL/min, and the injector temperature was maintained at 250 °C. The oven temperature was initially set at 70 °C and subsequently increased to 240 °C at a rate of 5 °C/min. Before injection, the essential oil was diluted 1:100 (*v*/*v*) in n-hexane. Mass spectra were acquired in electron-ionization mode at 70 eV over an *m*/*z* range of 35–425. The chemical compounds of the essential oil were identified by comparing their RI to those of n-alkanes (C8-C22) as external references, their retention times (RT), and their mass spectra with those reported in MS libraries (Wiley) [30].

### 4.3. Exploratory In Silico Physicochemical and ADMET Analysis

In silico profiling of pharmacokinetic and toxicological properties was performed for the major constituents selected as chemical markers of the essential oil (carvone, limonene, 1,8-cineole/eucalyptol, β-myrcene), together with the reference compounds used in the in vivo model (GAL and SCO). Chemical structures were retrieved as canonical/isomeric SMILES (Table 9) from PubChem (National Center for Biotechnology Information) (https://pubchem.ncbi.nlm.nih.gov/) and visually inspected to confirm structural correctness and, where applicable, stereochemistry, except for trans-dihydrocarvone, which was not available; therefore, its SMILES structure was generated manually.

Physicochemical descriptors, drug-likeness filters (Lipinski, Ghose, Veber, Egan, and Muegge), medicinal chemistry alerts, and qualitative predictions of gastrointestinal absorption and BBB permeability using the BOILED-Egg model were obtained with SwissADME [17] (https://www.swissadme.ch/) using default parameters [31,32].

Complementary ADMET endpoints (absorption, distribution, metabolism, and excretion), as well as selected safety signals (e.g., predictors of hERG-related risk and mutagenicity, where available), were generated using ADMET-AI [18] (https://admet.ai.greenstonebio.com/).

All outputs produced by these web servers were collected and processed between 20–30 December 2025 and exported to spreadsheets for comparative analysis and graphical representation. The calculations were treated as a post hoc exploratory analysis after evaluation of the experimental endpoints. WLOGP and TPSA were interpreted jointly in the BOILED-Egg model, and no fixed lipophilicity threshold was used as a stand-alone criterion for BBB penetration. No plasma, whole-body, or brain concentrations of the MEO constituents were measured in this study, and no experimental BBB/permeability assay was performed. Therefore, SwissADME and ADMET-AI outputs were used only as computational context and not as evidence of absorption, BBB passage, brain exposure, pharmacokinetics, or safety [21,32].

### 4.4. Animals, Housing Conditions, and Treatment Protocols

Adult zebrafish (*D. rerio*), Tübingen wild-type line, 4–6 months old, of both sexes, with a mean body weight of 0.35–0.45 g and a standard length of 3–4 cm, were obtained from the European Zebrafish Resource Center (EZRC, Karlsruhe Institute of Technology, Germany). Before the start of the experimental procedures, fish were left to acclimate for two weeks in the laboratory facility. They were housed in 10 L glass tanks under aerated conditions, maintained at a stable temperature of 26 ± 1 °C, with a controlled photoperiod of 14 h light and 10 h darkness. Water quality was checked daily and kept within optimal parameters (pH 7.0–7.5, dissolved oxygen 6.5–7.5 mg/L, conductivity 450–500 µS/cm). Fish were fed twice per day with commercial flake food (TetraMin, Tetra GmbH, Melle, Germany), but food was withdrawn 24 h before behavioral testing to avoid potential metabolic interference [21].

**Figure 9:**
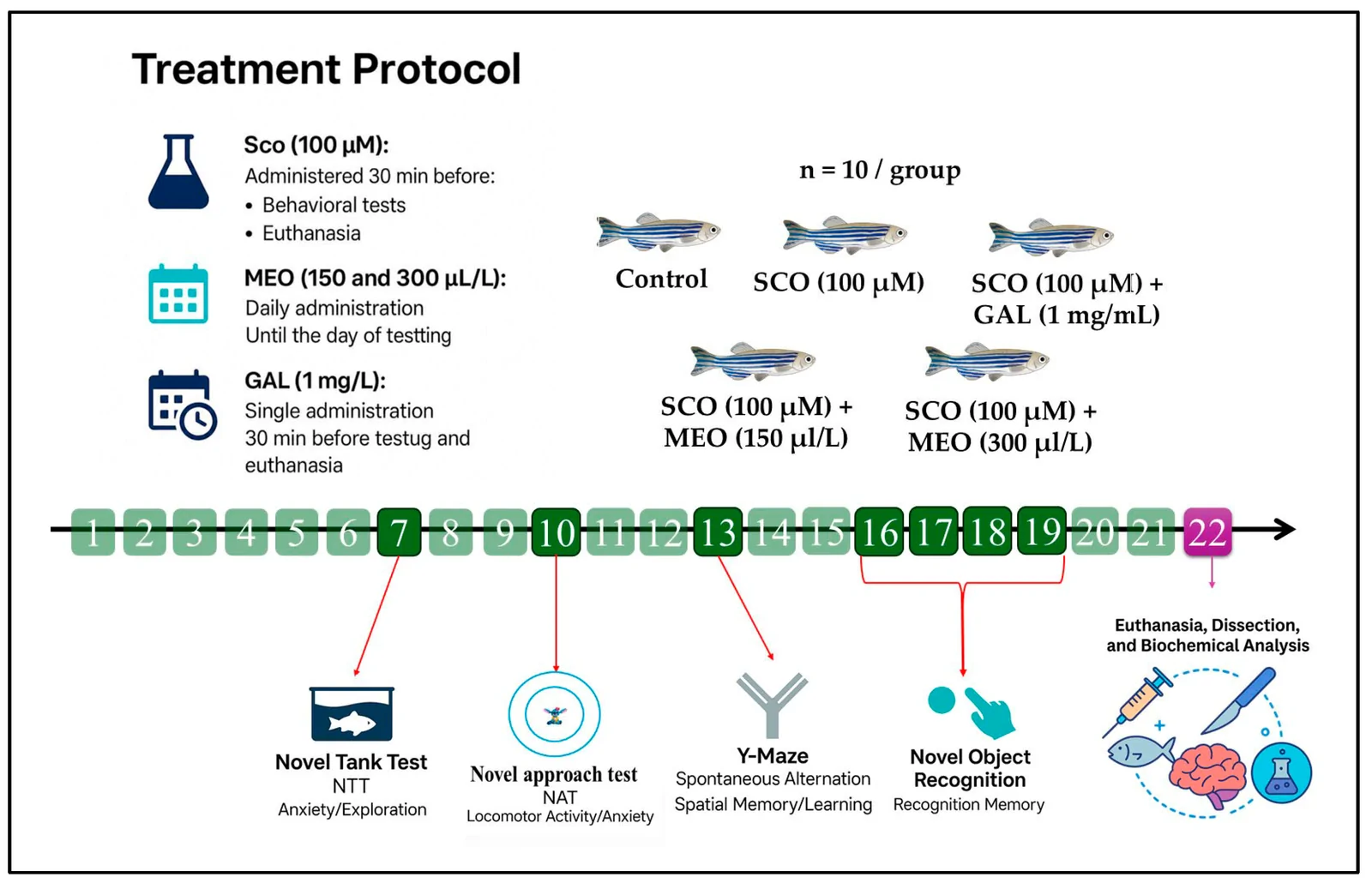
Experimental design and treatment chronology. MEO or vehicle exposure was renewed daily from experimental day 1 through day 22. Behavioral testing followed a fixed sequence: NTT on day 7, NAT on day 10, Y-maze on day 13, and NOR on days 17–20 (task-relative NOR habituation days 1–3 correspond to experimental days 17–19; training/testing correspond to experimental day 20). SCO (100 µM, 30 min) was administered immediately before each behavioral assessment and again before euthanasia on day 22. GAL (1 mg/L, 30 min) preceded each SCO challenge in the positive-control group. Brain tissue was collected on day 22. Each treatment condition used one 10 L tank with 10 fish. In the timeline, light-green boxes indicate days of ongoing MEO/vehicle exposure, dark-green boxes indicate behavioral-testing days, and the magenta box indicates euthanasia, tissue collection, and biochemical analysis on day 22.

Before allocation, fish were selected to be comparable in body size, apparent health, physical condition, and general activity. Sex was determined for all animals. Fish were then manually randomized among five groups (10 fish/group) while maintaining an equal sex distribution (5 males and 5 females/group). Each treatment group was maintained in one 10 L exposure tank. Exposure and water renewal occurred collectively at tank level, and treatment solutions were prepared separately for each group. Consequently, the chronic exposure condition had one tank-level replicate per treatment; individual fish were tested behaviorally but were not independent chronic-exposure replicates. Two separate MEO stock dispersions were prepared for the nominal 150 and 300 µL/L treatments. MEO was first dispersed in Tween 20 and then diluted with water; the final Tween 20 concentration was 0.02% in every experimental group, including Control, SCO, and GAL+SCO. Fresh treatment was applied daily during water renewal. The nominal MEO concentrations were not analytically verified in tank water, and no dedicated physicochemical stability study of the Tween 20 formulation was performed. Fish were housed in aerated 10 L tanks at 26 ± 1 °C under a 14 h light/10 h dark photoperiod and were fed twice daily except during the 24 h fasting period before behavioral testing. No mortality or overt adverse behavior requiring removal was observed [32,33]. MEO or vehicle exposure began on experimental day 1 and continued daily through day 22 (Figure 9). NTT was performed on day 7, NAT on day 10, Y-maze on day 13, and NOR on experimental days 17–20. SCO (100 µM) was administered for 30 min immediately before each relevant behavioral assessment and again before euthanasia on day 22. In the GAL+SCO group, GAL (1 mg/L) was administered for 30 min before each subsequent SCO challenge. After testing, fish were returned to their assigned housing/treatment conditions; daily MEO/vehicle exposure continued between paradigms, which were separated by at least 48 h of recovery.

Behavioral assessments were conducted individually between 08:00 and 17:00 under standardized environmental conditions. Testing order was interleaved across experimental groups throughout the day to reduce systematic order and circadian effects. Investigators conducting behavioral testing and video analysis were blinded to treatment allocation. The investigator preparing and administering treatment solutions necessarily knew group allocation. Video files were recorded with a Logitech C922 Pro HD webcam and analyzed with ANY-maze v7.48 (Stoelting CO., Wood Dale, IL, USA). Biochemical tubes were coded so that treatment identity was concealed during assay procedures and was resolved for the final statistical analysis after data acquisition.

All procedures complied with Directive 2010/63/EU and were approved by the Institutional Ethics Committee of Alexandru Ioan Cuza University, Iasi, Romania (BIO-UAIC-1714; approved 6 July 2023). Ice-cold-water immersion (2–4 °C) until cessation of opercular movements, followed by rapid decapitation, was specifically covered by the approved protocol and institutional zebrafish welfare procedures. Animals were monitored throughout the experiment for mortality, abnormal swimming, loss of equilibrium, feeding impairment, respiratory distress, and other overt distress. No mortality or adverse event requiring premature removal or euthanasia was observed. Animal work was conducted from September to October 2025.

### 4.5. Behavioral Testing

The same cohort completed the behavioral battery in a fixed sequence (NTT, NAT, Y-maze, NOR), identical for all groups. Successive paradigms were separated by at least 48 h of recovery. After each fish, the apparatus was completely emptied, thoroughly rinsed with clean water, then rinsed with distilled water and refilled with water from the housing system before the next animal was tested. These procedures standardized testing conditions, although residual repeated-testing effects cannot be completely excluded. GAL (1 mg/L) served as a pharmacological positive control rather than an intrinsic anxiolytic reference [34].

#### 4.5.1. Novel Tank Diving Test (NTT)

Anxiety-like behavior was first examined with the NTT [35], which takes advantage of the zebrafish tendency to remain near the bottom of a new environment when stressed. Each fish was gently placed in a trapezoid-shaped glass tank filled with fresh water, and its behavior was recorded for 6 min. To simplify the analysis, the tank was virtually divided into two equal zones (top and bottom). The following variables were quantified: latency to enter the top zone, time spent in the top/bottom ratio, distance traveled in the top/bottom ratio, number of entries to the top zone, total distance traveled, freezing duration, and average swimming speed (velocity).

#### 4.5.2. Novel Approach Test (NAT)

Locomotor and exploratory behaviors were further assessed using NAT [36]. The apparatus consisted of a circular opaque tank (diameter 34 cm, height 15 cm) filled with 6 cm of water kept at 25–28 °C. A multicolored Lego block (5 cm) was placed in the center of the arena to act as a novel object. Each fish was introduced facing the object and allowed to explore for 5 min. For video analysis, the arena was virtually divided into two zones: a central circular area (10 cm diameter) surrounding the object and an outer thigmotaxis zone near the walls. Main variables included latency to initiate exploration, total distance traveled, time in outer zone, and time in inner zone.

#### 4.5.3. Y-Maze Test

Spatial working memory was examined in a Y-shaped glass maze [37] with three arms of equal size (25 × 8 × 15 cm). To aid orientation, simple geometric patterns (squares, circles, triangles) were attached to the outer walls of each arm as visual cues. The test consisted of two trials separated by a 1 h interval. During the first trial, the fish could explore only two arms (start and open), while the third arm (novel) was blocked. In the second trial, access was granted to all arms. Behavioral endpoints included the percentage of spontaneous alternations and time spent in the novel arm as indices of short-term spatial memory and response to novelty, along with the number of arm entries, total distance traveled, turn angle, and number of line crossings as indicators of locomotor activity.

#### 4.5.4. Novel Object Recognition Test (NOR)

Recognition memory was assessed with the NOR task [38], in a transparent 30 × 30 × 30 cm tank containing 6 cm of water. The NOR-specific “days 1–3” refer to habituation phases and correspond to experimental days 17–19; NOR task day 4 corresponds to experimental day 20. During habituation, fish were placed in the empty tank for 5 min daily. On experimental day 20, two identical objects were introduced for a 10 min training phase; after a 1 h retention interval, one object was replaced with a novel object and exploration was recorded for 10 min. A preference index (%) was calculated as: [time spent with the novel object/(time spent with familiar (F) + novel object (N))] × 100. A higher index indicated intact recognition memory.

### 4.6. Biochemical Analysis

#### 4.6.1. Preparation of Enzyme Homogenates

After completion of behavioral assessments, zebrafish were euthanized by immersion in ice-cold water (2–4 °C) until opercular movements ceased, followed by rapid decapitation under the approved protocol. Brains were immediately extracted, weighed individually (≈3–6 mg), and stored at −20 °C until further analysis. The following day, tissues were homogenized at a 1:10 (*w*/*v*) ratio using ice-cold 0.1 M potassium phosphate buffer (pH 7.4) containing 1.15% KCl. Homogenization was performed with a bead-mill homogenizer (Mikro-Dismembrator U, Sartorius, Göttingen, Germany), and crude homogenates were centrifuged at 20,200× *g* for 15 min at 4 °C (Eppendorf 5417R, Hamburg, Germany). Supernatants were kept on ice and analyzed without prolonged storage. Samples were coded before biochemical analysis so that assay operators were blinded to treatment identity. Each assay included reagent blanks and technical duplicate readings; duplicates were averaged within each individual brain, and protein normalization was applied consistently across groups.

#### 4.6.2. Acetylcholinesterase (AChE) Activity

AChE activity in brain supernatants was determined spectrophotometrically by Ellman’s method [39], using acetylthiocholine iodide as substrate and DTNB as chromogenic reagent (Sigma-Aldrich, Darmstadt, Germany). Hydrolysis of ATCh releases thiocholine, which reacts with DTNB to yield a yellow-colored product detectable at 412 nm. Enzyme activity was expressed as nmol substrate hydrolyzed per min per mg protein. Protein concentration was determined by the Bradford assay [40].

#### 4.6.3. Antioxidant Enzyme Activities and Oxidative Stress Markers

The evaluation of antioxidant enzyme activities and oxidative stress markers was carried out as previously described in detail [41], ensuring reproducibility and comparability with our earlier reports. All determinations were normalized to protein content, quantified by the Bradford assay [40], and expressed as mean values from duplicate measurements. In summary, the enzymatic antioxidant defense was assessed by measuring SOD, CAT, and GPX activities. SOD activity [42] was based on the inhibition of NBT reduction in a riboflavin-light system, read at 560 nm. CAT activity [43] was determined by monitoring the decomposition rate of H_2_O_2_ and expressed as µmol H_2_O_2_ degraded/min/mg protein. GPX activity [44] was measured through oxidation of reduced GSH in the presence of H_2_O_2_, with residual GSH quantified using DTNB at 412 nm.

Non-enzymatic parameters were also analyzed. Reduced GSH [45,46] was quantified spectrophotometrically with DTNB at 412 nm, expressed as µmol/mg protein. Lipid peroxidation was assessed by determining MDA levels via the TBARS method [47], measured at 532 nm and expressed as nmol/mg protein. Protein carbonylation was quantified by the DNPH method [48], with absorbance at 370 nm, expressed as nmol DNPH/mg protein.

### 4.7. Statistical Analysis

No formal *a priori* power calculation was performed. The behavioral sample size of n = 10 fish/group was selected from previous zebrafish studies using comparable behavioral paradigms and treatment designs while considering the 3R principle. Biochemical analyses used n = 3 independent individual brains/group; technical duplicates were averaged within each brain and did not increase biological n. For each endpoint, the five treatment groups were compared by one-way ANOVA followed, after a significant omnibus test, by Tukey-adjusted pairwise comparisons. Because formal normality and homoscedasticity tests have very low diagnostic power at n = 3/group, Shapiro–Wilk/Brown–Forsythe/Bartlett results were interpreted cautiously rather than as confirmation of assumptions. Residual-versus-fitted and Q-Q plots for the seven biochemical endpoints are provided in Supplementary Figure S1. Exact *p*-values, effect-size estimates, and 95% confidence intervals are provided in Supplementary Data S1. As a distribution-free sensitivity analysis, permutation-based one-way tests were also performed for all seven biochemical endpoints; the overall treatment effect remained significant for each endpoint. In Supplementary Data S1, raw biochemical observations are reported in Biochemistry_raw, omnibus and FDR-adjusted statistics in Omnibus_stats, and complete Tukey-adjusted pairwise comparisons in Tukey_posthoc.

Multiplicity was addressed by applying Benjamini–Hochberg FDR correction separately to the behavioral and biochemical omnibus endpoint families; Tukey adjustment was retained for post hoc pairwise comparisons after significant omnibus tests. No single primary endpoint was formally preregistered, although the task-specific endpoints were defined before data analysis. Behavior–biomarker analyses used 15 same-fish paired observations (three fish/group) and were reanalyzed using linear regression with treatment group entered as a categorical covariate. Benjamini–Hochberg correction was then applied across the seven treatment-adjusted association tests. These analyses were designated exploratory and associative. Statistical analyses were conducted using GraphPad Prism v9.5 with complementary reanalysis scripts for FDR, treatment-adjusted associations, and permutation sensitivity calculations. The same-fish paired values and complete treatment-adjusted correlation outputs are provided in Supplementary Data S1 (sheets Correlation_paired and Correlation_stats, respectively).

## 5. Conclusions

This study shows that chronic immersion with MEO (150 and 300 μL/L), characterized by a carvone/limonene chemotype, mitigates SCO-induced anxiety-like behavior, cognitive impairment, and brain oxidative imbalance in adult zebrafish. Across behavioral paradigms, MEO reduced several anxiety-related endpoints in the NTT and NAT and improved cognitive performance in the Y-maze and NOR, although reduced distance traveled at 300 μL/L in selected assays indicates that locomotor effects should be considered when interpreting the higher concentration. At the biochemical level, MEO restored antioxidant defenses (SOD, CAT, and GPX) and GSH and reduced oxidative damage markers, with near-complete normalization of protein carbonyls and a partial reduction in lipid peroxidation (MDA), more evident at 300 μL/L. Correlation analyses were consistent with associations between cholinergic/redox status and behavioral performance but do not establish causality. The lower concentration (150 μL/L) yielded the clearest procognitive profile with less locomotor interference, whereas the higher concentration (300 μL/L) produced stronger normalization of selected oxidative markers but also showed a mild hypoactive component in some exploration-dependent tasks. These findings indicate endpoint-dependent optimal effects rather than a simple dose–response relationship. Computational profiling placed several major monoterpenes in physicochemical regions compatible with passive permeability; however, these model outputs do not demonstrate BBB passage or brain exposure under the immersion protocol. Collectively, the experimental findings support carvone-dominant MEO as a candidate for further preclinical investigation rather than as a validated neuroprotective adjunct. Future studies should identify the bioactive contributors within the mixture, directly quantify internal and brain exposure, characterize pharmacokinetics, extend the dose–response range and exposure duration, and evaluate potential sex-specific effects.

## Figures and Tables

**Table 1:** Chemical composition of *Mentha spicata* essential oil (MEO) as determined by GC-MS/GC-FID. Retention time, compound assignment, molecular formula, and relative peak-area percentage are reported for 12 constituents accounting for approximately 93.4% of the summed relative peak area. Percentages are semiquantitative relative peak areas rather than absolute concentrations.

Retention Time (min)	Compound	Molecular Formula	Percentage (% Area)
6.5	α-Pinene	C_10_H_16_	0.6
7.1	β-Myrcene	C_10_H_16_	1.8
8.4	Limonene	C_10_H_16_	10.6
9.2	1,8-Cineole (Eucalyptol)	C_10_H_18_O	4.2
11.7	trans-Dihydrocarvone	C_10_H_16_O	2.1
12.4	cis-Dihydrocarvone	C_10_H_16_O	1.2
13.6	Carvone	C_10_H_14_O	67.8
14.0	cis-Carvone oxide	C_10_H_14_O_2_	1.5
14.8	Carveol (isomer mixture)	C_10_H_16_O_2_	1.4
16.2	β-Caryophyllene	C_15_H_24_	1.0
17.0	Germacrene D	C_15_H_24_	0.7
18.3	Bicyclogermacrene	C_15_H_24_	0.5

**Table 2:** SwissADME-predicted physicochemical properties and characteristics of selected major *Mentha spicata* essential oil (MEO) markers and reference compounds. Reported descriptors include molecular weight (MW), topological polar surface area (TPSA), H-bond acceptors/donors (HBA/HBD), fraction Csp^3^ and number of rotatable bonds.

Compound/ Physicochemical Properties	Carvone	Limonene	1,8-Cineole (Eucalyptol)	Trans- Dihydrocarvone	β-Myrcene	GAL	SCO
Molecular weight (g/mol)	150.22	136.23	154.25	152.23	136.23	287.35	438.31
No. heavy atoms	11	10	11	11	10	21	26
No. arom. heavy atoms	0	0	0	0	0	6	6
Fraction Csp3	0.50	0.60	1.00	0.70	0.40	0.53	0.59
No. rotatable bonds	1	1	0	1	4	1	5
No. H-bond acceptors	1	0	1	1	0	4	8
No. H-bond donors	0	0	0	0	0	1	4
Molar refractivity	47.32	47.12	47.12	47.80	48.76	84.05	102.66
TPSA (Å^2^)	17.07	0.00	9.23	17.07	0.00	41.93	89.99

**Table 3:** SwissADME-predicted drug-likeness characteristics of selected major *Mentha spicata* essential-oil (MEO) markers and reference compounds. Reported descriptors include compliance with common drug-likeness filters (Lipinski, Ghose, Veber, Egan, Muegge), together with the bioavailability score.

Compound/Drug-Likeness	Carvone	Limonene	1,8-Cineole (Eucalyptol)	Trans- Dihydrocarvone	β-Myrcene	GAL	SCO
Lipinski	Yes; 0 violation	Yes; 0 violation	Yes; 0 violation	Yes; 0 violations	Yes; 0 violation	Yes; 0 violation	Yes; 0 violation
Ghose	No; 1 violation: MW < 160	No; 1 violation: MW < 160	No; 1 violation: MW < 160	No; 1 violation: MW < 160	No; 1 violation: MW < 160	Yes	Yes
Veber	Yes	Yes	Yes	Yes	Yes	Yes	Yes
Egan	Yes	Yes	Yes	Yes	Yes	Yes	Yes
Muegge	No; 2 violations: MW < 200, Heteroatoms < 2	No; 2 violations: MW < 200, Heteroatoms < 2	No; 2 violations: MW < 200, Heteroatoms < 2	No; 2 violations: MW < 200, Heteroatoms < 2	No; 2 violations: MW < 200, Heteroatoms < 2	Yes	Yes
Bioavailability Score	0.55	0.55	0.55	0.55	0.55	0.55	0.55

**Table 4:** Predicted absorption-related ADMET endpoints for selected essential-oil markers and reference compounds (ADMET-AI). Endpoints include human intestinal absorption (HIA), oral bioavailability probability, cell effective permeability (log (10^−6^ cm/s)), PAMPA permeability, and P-glycoprotein (P-gp) inhibition probability.

Compound/Absorption	Carvone	Limonene	1,8-Cineole (Eucalyptol)	Trans- Dihydrocarvone	β-Myrcene	GAL	SCO
Human Intestinal Absorption	1.00	1.00	1.00	1.00	1.00	1.00	0.87
Oral Bioavailability	0.89	0.84	0.98	0.92	0.71	0.76	0.75
Cell Effective Permeability (log(10^−6^ cm/s))	−4.06	−4.21	−4.13	−3.97	−4.39	−4.62	−5.71
PAMPA Permeability	0.98	0.98	1.00	0.99	0.96	0.91	0.47
P-glycoprotein Inhibition	0.02	0.01	0.01	0.01	0.04	0.29	0.07

**Table 5:** Predicted distribution-related endpoints (ADMET-AI). Blood–brain barrier (BBB) penetration probability, plasma protein binding rate (PPB, %), and volume of distribution at steady state (VDss, L/kg) are reported.

Compound/Distribution	Carvone	Limonene	1,8-Cineole (Eucalyptol)	Trans- Dihydrocarvone	β-Myrcene	GAL	SCO
Blood–Brain Barrier Penetration	0.99	1.00	1.00	0.99	0.99	0.96	0.35
Plasma Protein Binding Rate (%)	61.33	83.97	56.64	60.64	91.14	44.34	48.16
Volume of Distribution at Steady State (L/kg)	0.00	3.53	1.47	0.00	9.12	2.00	4.06

**Table 6:** Predicted metabolism endpoints (ADMET-AI). Metabolic endpoints include predicted inhibition probabilities for CYP1A2, CYP2C19, CYP2C9, CYP2D6, and CYP3A4, as well as predicted substrate likelihoods (where reported).

Compound/Metabolism	Carvone	Limonene	1,8-Cineole (Eucalyptol)	Trans- Dihydrocarvone	β-Myrcene	GAL	SCO
CYP1A2 Inhibition	0.13	0.15	4.16 × 10^−3^	0.04	0.32	0.09	1.64 × 10^−3^
CYP2C19 Inhibition	0.22	0.26	0.26	0.12	0.38	0.16	0.05
CYP2C9 Inhibition	0.02	0.01	0.02	0.01	0.07	0.01	4.56 × 10^−3^
CYP2D6 Inhibition	2.64 × 10^−3^	0.02	2.05 × 10^−3^	2.03 × 10^−3^	0.01	0.54	0.04
CYP3A4 Inhibition	3.71 × 10^−3^	2.97 × 10^−3^	1.35 × 10^−3^	1.48 × 10^−3^	0.01	0.02	0.01
CYP2C9 Substrate	0.31	0.26	0.14	0.34	0.08	0.17	0.03
CYP2D6 Substrate	0.32	0.47	0.32	0.38	0.20	0.76	0.10
CYP3A4 Substrate	0.55	0.42	0.54	0.54	0.28	0.69	0.34

**Table 7:** Excretion endpoints (ADMET-AI) include half-life (h) and predicted clearance in hepatocytes (µL/min/10^6^ cells) and microsomes (µL/min/mg).

Compound/ Excretion	Carvone	Limonene	1,8-Cineole (Eucalyptol)	Trans- Dihydrocarvone	β-Myrcene	GAL	SCO
Half Life (h)	0.00	2.69	2.95	0.00	10.14	13.94	38.87
Drug Clearance (Hepatocyte) (µL/min/10^6^ cells)	59.37	72.19	37.73	77.73	101.60	15.99	37.20
Drug Clearance (Microsome) (µL/min/mg)	18.10	38.32	2.60	29.38	52.40	0.00	0.00

**Table 8:** Predicted toxicity endpoints and pathway-associated signals (ADMET-AI). The table includes predicted hERG blocking probability, clinical toxicity, mutagenicity, drug-induced liver injury (DILI), carcinogenicity, acute toxicity (LD50-related metric), skin reaction, and selected mechanistic pathway signals (e.g., NRF2/ARE), reported as platform outputs.

Compound/ Toxicity	Carvone	Limonene	1,8-Cineole (Eucalyptol)	Trans- Dihydrocarvone	β-Myrcene	GAL	SCOP
hERG Blocking	0.01	0.10	0.20	0.01	0.10	0.61	0.49
Clinical Toxicity	0.01	0.01	2.38 × 10^−3^	0.01	0.03	0.29	0.45
Mutagenicity	0.02	0.01	0.11	0.01	0.10	0.44	0.37
Drug-Induced Liver Injury	0.18	0.04	0.14	0.12	0.12	0.07	0.09
Carcinogenicity	0.09	0.13	0.13	0.06	0.58	0.02	0.04
Acute Toxicity LD50 (log(1/(mol/kg)))	2.97	1.66	1.66	2.30	1.19	3.27	2.43
Skin Reaction	0.66	0.65	0.63	0.41	0.95	0.55	0.52
PPARγ (Peroxisome Proliferator-Activated Receptor Gamma)	7.18 × 10^−4^	3.43 × 10^−4^	2.23 × 10^−4^	7.29 × 10^−4^	0.01	3.63 × 10^−3^	3.58 × 10^−3^
NRF2/ARE (Nuclear Factor (Erythroid-Derived 2)-Like 2/Antioxidant Responsive Element)	0.03	0.01	0.01	0.02	0.42	0.12	0.04
ATAD5	2.82 × 10^−4^	3.22 × 10^−5^	1.26 × 10^−4^	9.49 × 10^−5^	1.56 × 10^−3^	0.01	0.01
Heat Shock Factor Response Element	0.01	0.01	0.01	0.01	0.32	0.02	0.01
Mitochondrial Membrane Potential	2.87 × 10^−3^	0.01	0.03	3.84 × 10^−3^	0.02	0.07	0.01
Tumor Protein p53	3.57 × 10^−4^	1.20 × 10^−4^	1.16 × 10^−4^	2.30 × 10^−4^	4.85 × 10^−3^	0.01	0.03

**Table 9:** SMILES strings of volatile constituents identified by GC-MS and reference compounds used for in silico ADMET analyses. Canonical/isomeric SMILES were retrieved from PubChem (accessed 20–22 December 2025) and visually checked for structural correctness and stereochemistry when applicable. Trans-dihydrocarvone was not available in PubChem at the time of retrieval, and its SMILES string was generated manually.

Compound	SMILES
α-Pinene	CC1=CCC2CC1C2(C)C
β-Myrcene	CC(=CCCC(=C)C=C)C
Limonene	CC1=CC[C@H](CC1)C(=C)C
1,8-Cineole (Eucalyptol)	CC1(C2CCC(O1)(CC2)C)C
trans-Dihydrocarvone	C=C(C)C1CCC(C)C(=O)C1
cis-Dihydrocarvone	C[C@@H]1CC[C@@H](CC1=O)C(=C)C
Carvone	CC1=CCC(CC1=O)C(=C)C
cis-Carvone oxide	C[C@H]1CC[C@@]2([C@H](C1=O)O2)C(C)C
Carveol (isomer mixture)	CC1=CCC(CC1O)C(=C)C
β-Caryophyllene	C/C/1=C\CCC(=C)[C@@H]2CC([C@H]2CC1)(C)C
Germacrene D	C/C/1=C\CCC(=C)/C=C/[C@@H](CC1)C(C)C
Bicyclogermacrene	C/C/1=C\CC/C(=C/[C@H]2[C@H](C2(C)C)CC1)/C
Galantamine	CN1CC[C@@]23C=C[C@@H](C[C@@H]2OC4= C(C=CC(=C34)C1)OC)O
Scopolamine hydrobromide trihydrate	CN1[C@@H]2CC(C[C@H]1[C@H]3[C@@H]2O3) OC(=O)[C@H](CO)C4=CC=CC=C4.O.O.O.Br

## Data Availability

Raw behavioral data, raw individual biochemical data, paired behavior-biochemistry data, omnibus statistics, treatment-adjusted correlation analyses, sensitivity analyses, and full post hoc outputs are provided in Supplementary_RawData.xlsx. Biochemical residual-versus-fitted and Q-Q diagnostic plots are provided as Supplementary Figure S1. GC chromatograms/original instrument files are not included in the present supplementary package and should be supplied by the corresponding author if available and requested.

## Supplementary Materials

The following supporting information can be downloaded at https://www.mdpi.com/article/10.3390/plants15182763/s1. Figure S1: Diagnostic plots for the biochemical one-way ANOVA models. Residual-versus-fitted plots and quantile–quantile (Q–Q) plots of model residuals are shown for acetylcholinesterase (AChE), superoxide dismutase (SOD), catalase (CAT), glutathione peroxidase (GPX), reduced glutathione (GSH), protein carbonyls (PC), and malondialdehyde (MDA); Supplementary Data S1: Raw individual behavioral data for the NTT, NAT, Y-maze, and NOR; raw individual biochemical data; paired behavior–biomarker data; omnibus statistics; treatment-adjusted correlation analyses; sensitivity analyses; and complete Tukey-adjusted post hoc comparisons.

## Abbreviations

The following abbreviations are used in this manuscript:

AD	Alzheimer’s disease
ACh	Acetylcholine
AChE	Acetylcholinesterase
ANOVA	Analysis of variance
ATCh	Acetylthiocholine
CAT	Catalase
CI	Confidence interval
DNPH	2,4-Dinitrophenylhydrazine
DTNB	5,5′-Dithiobis(2-nitrobenzoic acid)
FO	Familiar object
GAL	Galantamine
GC-MS	Gas chromatography–mass spectrometry
GPX	Glutathione peroxidase
GSH	Reduced glutathione
MDA	Malondialdehyde
MEO	*Mentha spicata* L. essential oil
NAT	Novel approach test
NO	Novel object
NOR	Novel object recognition
NTT	Novel tank diving test
PC	Protein carbonyls
r	Pearson correlation coefficient
R^2^	Coefficient of determination
SCO	Scopolamine hydrobromide trihydrate
SEM	Standard error of the mean
SOD	Superoxide dismutase
TBARS	Thiobarbituric acid reactive substances
T/B	Top/bottom ratio
TRP	Transient receptor potential
Y-maze	Y-shaped maze
