# Supplementary material for: Chronic Administration of Carvone-Rich Mentha spicata L. Essential Oil Attenuates Cognitive Dysfunction and Oxidative Stress in Zebrafish
Source: Plants (Basel). 2026 Sep 9;15(18):2763. doi: 10.3390/plants15182763 (PMC13610066; doi:10.3390/plants15182763)
Supplement: Supplementary file 1 [file plants-15-02763-s001.zip › Supplementary_Data_ Figure S1.pdf]

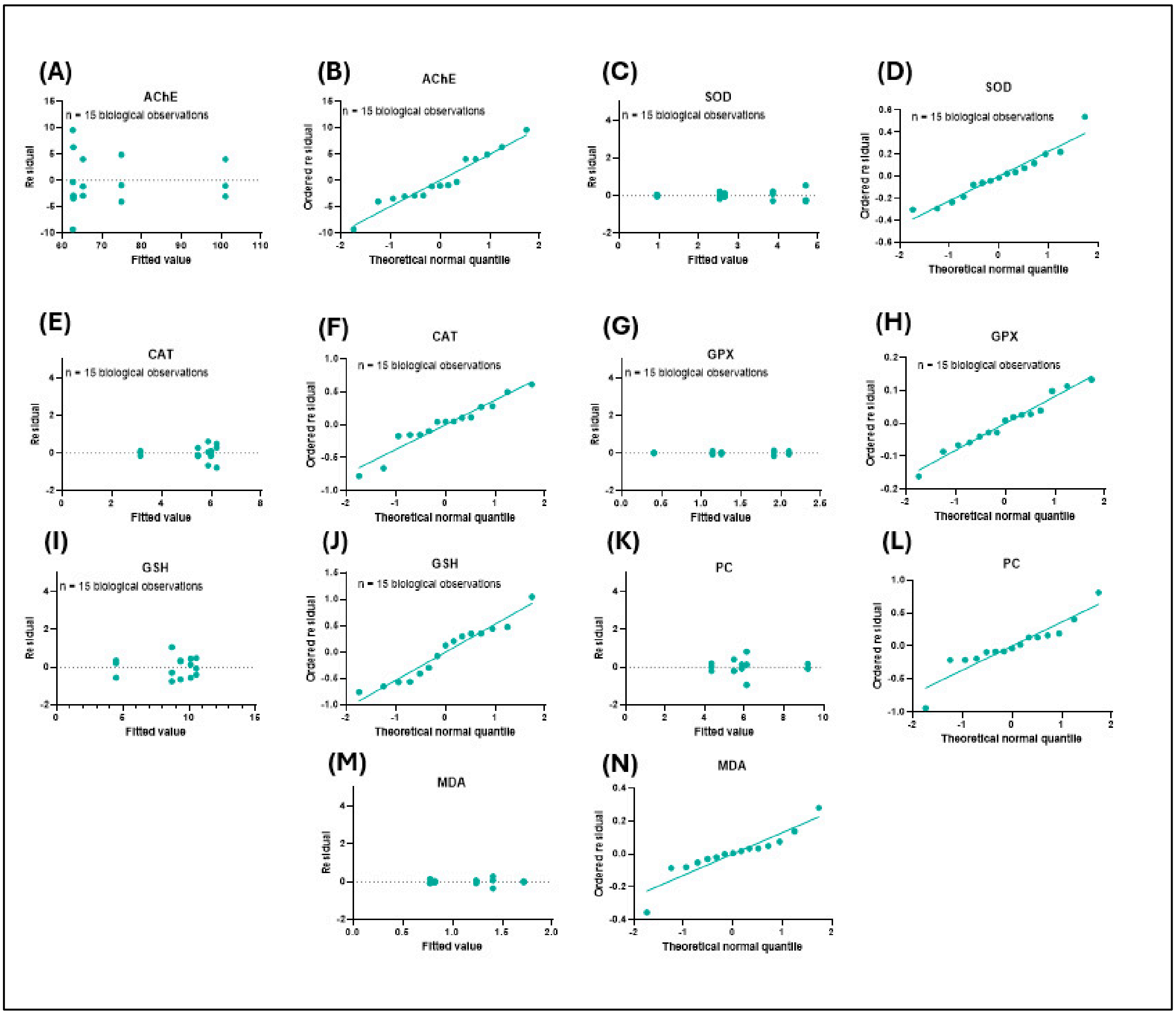

Figure S1. Diagnostic plots for the biochemical one-way ANOVA models. Residual-versus-fitted plots and quantile–quantile (Q–Q) plots of model residuals are shown for acetylcholinesterase (AChE; A,B), superoxide dismutase (SOD; C,D), catalase (CAT; E,F), glutathione peroxidase (GPX; G,H), reduced glutathione (GSH; I,J), protein carbonyls (PC; K,L), and malondialdehyde (MDA; M,N). Each analysis included 15 independent biological observations (n = 3 zebrafish brains per treatment group). Technical duplicate measurements were averaged before analysis and were not treated as independent observations. Diagnostic plots were interpreted cautiously because of the limited biological sample size.
